# Development of orthogonal NISTmAb size heterogeneity control methods

**DOI:** 10.1007/s00216-017-0819-3

**Published:** 2018-02-10

**Authors:** Abigail Turner, Katharina Yandrofski, Srivalli Telikepalli, Jason King, Alan Heckert, James Filliben, Dean Ripple, John E. Schiel

**Affiliations:** 1 National Institute of Standards and Technology, Institute for Bioscience and Biotechnology Research, 9600 Gudelsky Dr, Rockville, MD 20850 USA; 2 Present Address: MedImmune, LLC, 55 Watkins Mill Rd, Gaithersburg, MD 20878 USA; 3 National Institute of Standards and Technology, 100 Bureau Drive, Gaithersburg, MD 20899 USA

**Keywords:** Reference material, NISTmAb, Monoclonal antibody, Biotherapeutic, Biopharmaceutical, System suitability, Biosimilar, Capillary electrophoresis, Protein particle, Aggregation

## Abstract

The NISTmAb is a monoclonal antibody Reference Material from the National Institute of Standards and Technology; it is a class-representative IgG1κ intended to serve as a pre-competitive platform for harmonization and technology development in the biopharmaceutical industry. The publication series of which this paper is a part describes NIST’s overall control strategy to ensure NISTmAb quality and availability over its lifecycle. In this paper, the development of a control strategy for monitoring NISTmAb size heterogeneity is described. Optimization and qualification of size heterogeneity measurement spanning a broad size range are described, including capillary electrophoresis-sodium dodecyl sulfate (CE-SDS), size exclusion chromatography (SEC), dynamic light scattering (DLS), and flow imaging analysis. This paper is intended to provide relevant details of NIST’s size heterogeneity control strategy to facilitate implementation of the NISTmAb as a test molecule in the end user’s laboratory.

**Electronic supplementary material:**

The online version of this article (10.1007/s00216-017-0819-3) contains supplementary material, which is available to authorized users.

**Graphical abstract:**
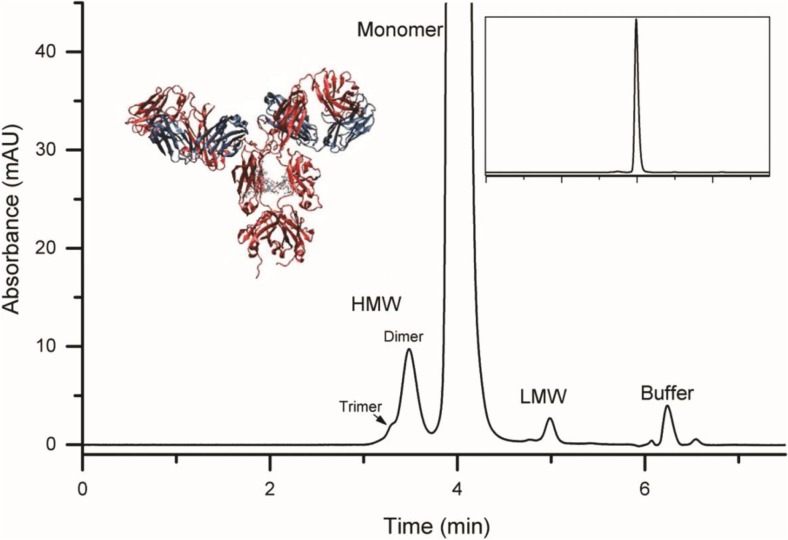
Representative size exclusion chromatogram of the NIST monoclonal antibody (NISTmAb). The NISTmAb is a publicly available research tool intended to facilitate advancement of biopharmaceutical analytics. HMW = high molecular weight (trimer and dimer), LMW = low molecular weight (2 fragment peaks). Peak labeled buffer is void volume of the column from L-histidine background buffer.

## Inroduction

Monoclonal antibody drug products are comprised of complex populations of structural variants which may differ in terms of size, charge, potency, and/or immunogenicity. This unavoidable complexity is derived from the biomanufacturing process [1] and in some cases may even be desirable. In every case, however, therapeutic mAb structural variation must be well defined, and its effects on safety and efficacy must be understood [2]. The burden, then, is on the analytical tools used to define mAb structure and function to provide a holistic definition of the mAb product that is supported by rigorous science. The intended purpose of NISTmAb RM 8671, the first monoclonal antibody Reference Material from the National Institute of Standards and Technology (NIST), is to provide a common platform for evaluating and understanding current (and emerging) state-of-the-art in mAb analysis technology, thus bolstering and streamlining therapeutic mAb characterization efforts. The NISTmAb has undergone extensive characterization through interlaboratory collaborations and in-house method development and quality control; these characterization efforts are ongoing and expanding to create an extensive knowledgebase surrounding the material [3–7]. The focus of this publication series is to describe the methods and control strategy NIST has implemented in-house for monitoring NISTmAb attributes over its life cycle. In this report, we discuss monitoring of NISTmAb size variants with emphasis on method development, qualification, and lifecycle appropriate implementation for longitudinal evaluation of RM quality attributes.

Evaluation of size heterogeneity is perhaps one of the most mechanistically complex and analytically daunting attributes to fully characterize [8–10]. Size heterogeneity encompasses a range of product-related species and/or impurities that may include small molecular weight sub-species (fragments) of the monomeric protein, oligomers on the order of 2–4 associated molecules, and highly multimeric protein particulates. These species may arise from chemical, conformational, and/or physical stress and encompass a size range of nanometers to hundreds of micrometers [9, 10]. Combined with the potential impact of size variants on potency and immunogenicity, it is no surprise that a variety of analytical and biophysical approaches have been developed to evaluate them.

The NISTmAb monomer consists of two heavy chains (≈50 kDa each) and two light chains (≈25 kDa each) connected by inter-chain disulfide linkages [11]. Low molecular weight (LMW) variants (fragments) may be generated non-enzymatically during manufacture, storage, and handling [12–16]—yielding some combination of heavy and light chains smaller than the monomer. Common mechanisms of mAb fragmentation include hydrolysis at low or high pH, disulfide scrambling, and ß-elimination at high pH [12]. The presence of fragments in mAb preparations is monitored by electrophoretic or chromatographic separations.

Size exclusion chromatography (SEC) is the method of choice for overall evaluation of size heterogeneity. SEC separates size variants (under non-denaturing conditions) using a porous-particle stationary phase that retains analytes based on their differential access to the pore volume [17–19]. SEC can be used to measure LMW variants, monomer, and high-molecular weight (HMW) variants in the same analysis yielding a measure of monomeric purity. Resolution of individual fragment species, however, is typically poor. Capillary electrophoresis-sodium dodecyl sulfate (CE-SDS) is commonly used to measure the overall fragmentation pattern and for quantification of purity [20–23]. CE-SDS is the capillary analog of SDS-PAGE, wherein the polyacrylamide slab gel is replaced with a narrow-bore glass capillary or microfabricated channel filled with a replaceable polymer sieving matrix which separates low molecular weight mAb variants and lower-order covalent aggregates from the mAb monomer. CE-SDS offers several advantages over SDS-PAGE, including on-column detection for facile quantification, high resolution, low sample consumption, reproducibility, automation and speed. CE-SDS is performed under denaturing conditions and is not amenable to sample collection for downstream analysis, SEC methods performed under non-denaturing conditions are therefore often used to collect fragments for further characterization.

HMW variants range from small soluble oligomers (dimer, trimer, tetramer) to larger nanometer aggregates and micrometer particles to very large visually observable particles. Aggregates reduce the concentration of efficacious drug and may be immunogenic [18, 24, 25]. The method chosen to monitor HMW species depends upon the size of the HMW variant of interest. SEC is run under approximately native conditions and it can be used to measure non-covalent aggregates up to ≈100 nm in diameter [18, 26]. With modern ultra-performance columns, good resolution of monomer from dimer, trimer, and tetramer is achieved in less than 15 min [27].

A relatively routine method for characterizing aggregation behavior of proteins in solution is dynamic light scattering (DLS), as it can size particles ranging from 1 nm to 1 μm in size. Particles moving in solution by Brownian motion scatter light; by analyzing the fluctuations in the intensity of the scattered light as a function of time, one can calculate the translational diffusion coefficient of the particles and consequently the hydrodynamic radius by knowing the viscosity and temperature of the solution. The correlation of intensity to time can be performed rapidly by a digital autocorrelator embedded in the DLS software. The size can then be obtained from the correlation function using different algorithms. One technique is to use a method of cumulants analysis to obtain the mean size, given by a Z-average diameter (referred to as the hydrodynamic diameter throughout this text), and an estimate of the width of the distribution, described by a polydispersity index (PDI) [28]. The samples, however, need to be homogenous with low polydispersity since size determination can be biased towards large particles even if only a few large particles are present.

Flow imaging (FI) is an optical technique that can be used to detect subvisible protein particles (> 1 μm in size). A high resolution digital camera captures and stores images of the particles present in the sample of interest as they pass through a flow cell of defined thickness.

Like its common orthogonal counterpart, the light obscuration method, FI can provide size distribution (size and concentration) data of the particles in the sample. In addition, FI offers the ability to perform morphological analysis using the software’s complex algorithm. Stored images of the particles can be retrieved and analyzed for various parameters such as aspect ratio, translucency, circularity, etc. Due to its improved sensitivity to translucent particles, the FI can better detect and more accurately size translucent proteinaceous particles and differentiate proteinaceous from non-proteinaceous particles than the light obscuration method.

In this report, we describe development and qualification of methods for routine monitoring of NISTmAb size variants: CE-SDS for LMW variants and SEC for HMW and LMW variants. The method development detailed here builds upon and further refines crowd-sourced methods for NISTmAb characterization documented in an ACS Symposium Series book compilation [8]. The qualified CE-SDS and SEC methods cover the range of mAb size variants up to ≈100 nm and are useful for monitoring NISTmAb structure, purity, and stability. We also describe a flow imaging method for measuring and characterizing subvisible particulates as well as the use of dynamic light scattering to evaluate the mean hydrodynamic diameter in the NISTmAb. Together, these methods are implemented to provide method performance criteria for the in-house primary sample 8670 (PS 8670) and further used as a system suitability control during value assignment ofNISTmAb RM 8671 size heterogeneity as described in ref. [29].

## Materials and methods

### CE-SDS materials and methods

#### Materials

Method development, optimization, and qualification were carried out on Primary Sample 8670 (PS 8670) derived from a single production lot of the NISTmAb. Bare fused silica capillaries (50 μm inner diameter, 67 cm total length) were purchased from Sciex Separations (PN 338451) and cut to 30.5 cm prior to use. SDS-MW gel buffer, acidic and basic wash solutions, MW ladder, 10 kDa internal standard, and Tris/SDS sample buffer (pH 9.0) were purchased as a kit from Sciex Separations (PN 390953). Citric acid (PN 27487-50G-F), sodium phosphate dibasic dihydrate (PN 71633-250G), pre-weighed iodoacetamide vials (PN A3221-10VL), 2-mercaptoethanol (PN M3148-25ML), and 20% (*w*/*v*) SDS solution (PN 05030–50 ± 0ML-F) were from Sigma Aldrich. L-histidine monohydrochloride (PN 2081–06) and L-histidine (PN 2080–05) were from J. T. Baker. Zeba columns used for buffer exchange were from Life Technologies (PN 89882). PVDF syringe filters were from EMD Millipore (PN SLSV025LS).

#### Instrumental method

All samples were analyzed using a Sciex Separations PA800 *plus* pharmaceutical analysis system. New capillaries (Sciex PN 338451, bare fused silica, 50 μm internal diameter × 30.5 cm) were conditioned using the method in Table S4 (see Electronic Supplementary Material, ESM). Samples were prepared as described in the ESM and analyzed using the method in Table S5 (see ESM). Samples were detected at the capillary window, 20 cm from the inlet, using the PA800 plus PDA detector set to collect absorbance at 220 nm. See Tables S7 and S8 in the ESM for instrument configuration details. At the end of each sequence, the capillary was prepared for storage using the method in Table S6 (see ESM), then stored at room temperature with the ends dipped in DIUF water. Injection sequences were of the form Blank─IQ─8670 × 3─IQ─Blank. Electropherograms were analyzed using the 32Karat software package (Sciex Separations) as described in the CIEF data analysis section of the ESM.

### SEC materials and methods

#### Materials

Method development, optimization, and qualification were carried out on Primary Sample 8670 (PS 8670) derived from a single production lot of the NISTmAb. Acquity UPLC Protein BEH SEC Column (1.7 μm particle size, 200 Å pore size, 4.6 mm × 150 mm length) was obtained from Waters Corporation (PN 186005225). Sodium phosphate monobasic (PN 17844-250G), Sodium phosphate dibasic dihydrate (PN 71633-250G), sodium chloride (73575-250G–F) were from Sigma Aldrich. IQ gel filtration standard #1 lot 220,004,819 and #2 lot 64,038,897 were from Bio-Rad (PN 151–1901). 0.22 μm cellulose acetate filters were from Corning (PN 430015). Vial inserts (PN 5182–0549), vials (PN 5182–0716), and vial caps (PN 5182–0717) were from Agilent Technologies.

#### Instrumental method

All samples were analyzed on a Thermo Scientific/Dionex U3000 high-pressure liquid chromatography system equipped with a Thermo Scientific Dionex flow cell (cell volume: 2.5 µL, path length: 7 mm, pressure limit: 12 MPa, PN 6074.0360). The SEC column was pre-conditioned with mobile phase for 30 min before sample analysis. Samples were injected (6 μL, except as noted for linearity and LOD/LOQ determination) into a pre-conditioned column using a flow rate of 0.300 mL/min, resulting in a pressure of ≈180 bar. Isocratic elution was monitored for each injection using a total run time of 10 min. Peaks were detected by ultraviolet absorbance using the variable wavelength detector at a wavelength of 280 nm. Raw chromatograms were processed with Thermo Scientific Dionex Chromeleon 7 Chromatography Data System as described in the ESM.

### DLS materials and methods

#### Materials

Method development, optimization, and qualification were carried out on Primary Sample 8670 (PS 8670) derived from a single production lot of the NISTmAb.

System suitability was performed using 200 nm diameter polystyrene microspheres lot 43,714 from Thermo Scientific (PN 3200A). A Malvern Zetasizer Nano ZS, Software version 7.11 was used for running the samples, which were contained in ZEN0040 disposable polystyrene micro-cuvettes (PN ZEN0040).

#### Instrumental method

At the beginning of each day of analysis, 70 μL of 200 nm diameter suspension of polystyrene microspheres in water was loaded into a clean ZEN0040 disposable cuvette and analyzed for size distribution to ensure that the sizing results of the microspheres were consistent with the intermediate precision values obtained during earlier analysis (data not shown). The instrument was equipped with backscatter detector fixed at a 173° angle, a built-in digital autocorrelator to calculate the autocorrelation function, and auto-attenuation capabilities. All samples were run in triplicates, unless noted otherwise, with each sample being acquired in 3 successive measurements, with 10 scans, and with an equilibration time of 60 s (*n* = 9). The viscosity of the sample was approximated to be the viscosity of water at 20 °C (1.002 mPa⋅s), and the refractive index of the polystyrene microspheres was estimated to be 1.59. For each run reported, the count rates were above 2×10^5^ s^−1^ with a polydispersity index (PDI), which is an indicator of the heterogeneity of the sample, below 0.04. Zetasizer software version 7.11 was used to calculate the hydrodynamic diameter of the particles in the solution; the intensity based values were used for analysis.

Prior to running the protein samples, each individual vial was gently inverted five times to ensure even mixing of the sample. PS 8670 was run using similar parameters as described above except the refractive index of the protein particles was approximated to be 1.41 [30]. These protein samples were measured directly without further dilution or any other preparation steps. PS 8670 was run a total of 8 times with three scans each (*n* = 24) over three days. Due to sample limitations, only inter-vial repeatability values are shown. In order to be included in the analysis, the protein samples had to show count rates above 2.5×10^5^ s^−1^ and a polydispersity index below 0.2 [31].

### Flow imaging materials and methods

#### Materials

Method development, optimization, and qualification were carried out on Primary Sample 8670 (PS 8670) derived from a single production lot of the NISTmAb. System suitability, repeatability, size, and count accuracy studies were performed with 5 μm diameter Count Precision Standards (Count-Cal microspheres) of nominal 3000 mL^−1^ concentration, 2 μm polystyrene (lot # 44870, PN 4202A), and 10 μm polystyrene microspheres (lot # 40284, PN 4210A) (Thermo Scientific) (all sizes reported as microsphere diameter). Repeatability assessments and method optimization experiments were performed using polyclonal human IgG purchased from Sigma (lot #082M4772V, PN I2511-10MG) and a partially fluorinated polymer called ethylene tetrafluorethylene (ETFE) that mimics the appearance of protein particles. The method to produce ETFE is described in Ripple et al. [32]. Briefly, ETFE tubing was abraded against a diamond abrasive disc. The particles were collected from the disc and diluted into a filtered 0.01% (*w*/*v*) sodium lauryl sulfate (Soap Goods, Sodium Lauryl Sulfate (SLS Liquid)) and 0.02% (w/v) sodium azide (Ricca Chemical Company, PN 7144–32) solution. The particles in solution were filtered through a 51 μm nylon filter. The filtrate was retained for the measurements.

Micro-Flow Imaging (FI) DPA4200 flow imaging instrument and MVSS V. 2 software (Protein Simple) was used for acquiring and exporting data. MVAS 1.4 software was used for data analysis along with Lumetics software (Version 1.3)*.* In-house software, using Python software, was designed for calculating the approximate mass of protein within protein particles in each of the protein samples.

Samples were loaded using Neptune 1 mL barrier pipette tips (PN BT1000.96.N). Prior to using the instrument, it is imperative to perform all of the required instrument checks and verify that all tubing is calibrated as described in the instrument’s instruction manual. On the day of analysis, several milliliters of particle free water DIUF water should be flushed through to obtain a particle-free baseline. If a particle-free baseline cannot be obtained, a rigorous cleaning procedure, described in the instruction manual needs to be applied.

Prior to each analysis, the flow cell’s optimization illumination was performed with water. All samples were gently inverted 5 times and 0.7 mL of each sample was removed using a low protein-binding pipette tip and loaded into the instrument’s sample holder. The flow cell was first primed with 0.2 mL of the sample before data acquisition to minimize dilution effects. The remaining sample flowed through at a rate of 0.17 mL/min with approximately 1760 frames being taken up over the course of the run. Between each run, 2 mL of particle-free water was primed through the instrument and buffer controls were analyzed to ensure no carryover of particles from the previous runs. Histidine buffers were also analyzed as controls. All PS 8670 samples were measured directly without further dilution or any other preparation steps.

## Results and discussion

All methods used to monitor NISTmAb size variants were optimized and, where applicable, qualified as fit for quality monitoring over the lifecycle of the material [33]. Method development, optimization, and qualification were carried out on Primary Sample 8670 (PS 8670) derived from a single production lot of the NISTmAb.

### Monitoring NISTmAb LMW variants with CE-SDS

**Fig. 1:**
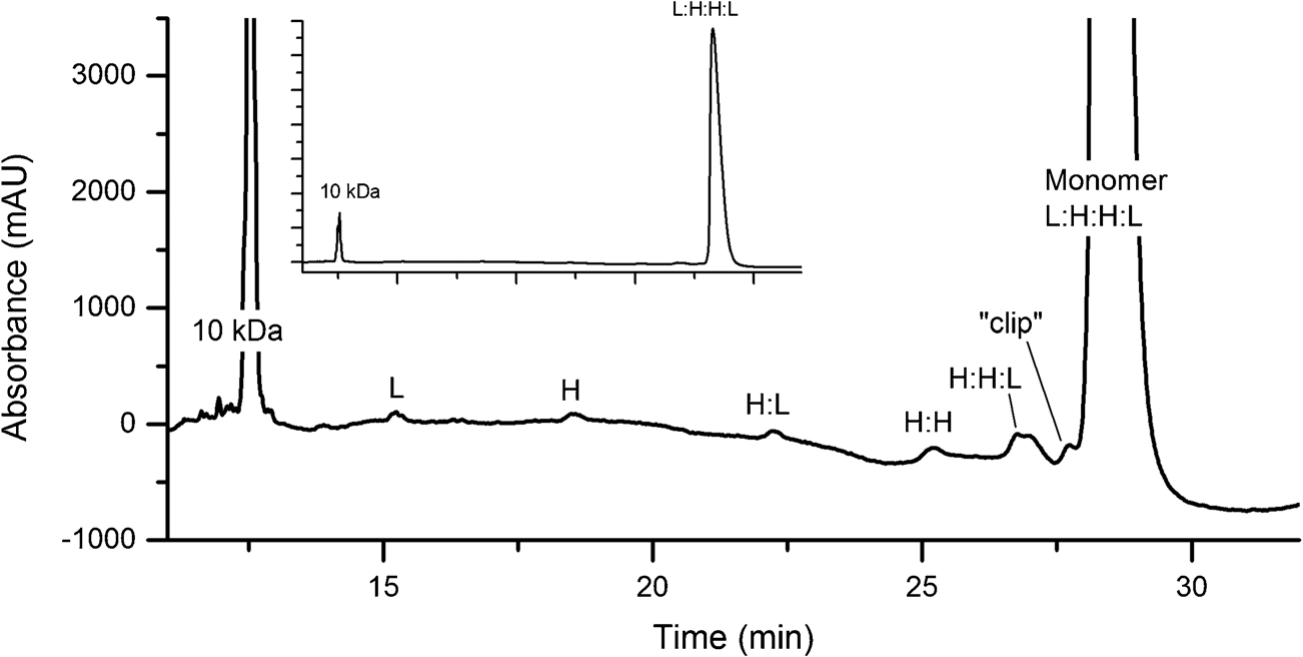
Representative electropherogram of PS 8670 analyzed by CE-SDS under non-reducing conditions. The inset trace is fully zoomed out to show the relative height of the monomer (major peak) relative to the smaller fragment peaks. No peaks were detected after the monomer peak. Putative peak assignments are based on migration times, but have not been evaluated based on orthogonal techniques. L = light chain; H = heavy chain; H:L = heavy chain:light chain fragment; H:H = heavy chain:heavy chain fragment; H:H:L = heavy chain:heavy chain:light chain fragment; L:H:H:L = monomer; clip = unidentified clip species of slightly lower molecular weight relative to the monomer

**Fig. 2:**
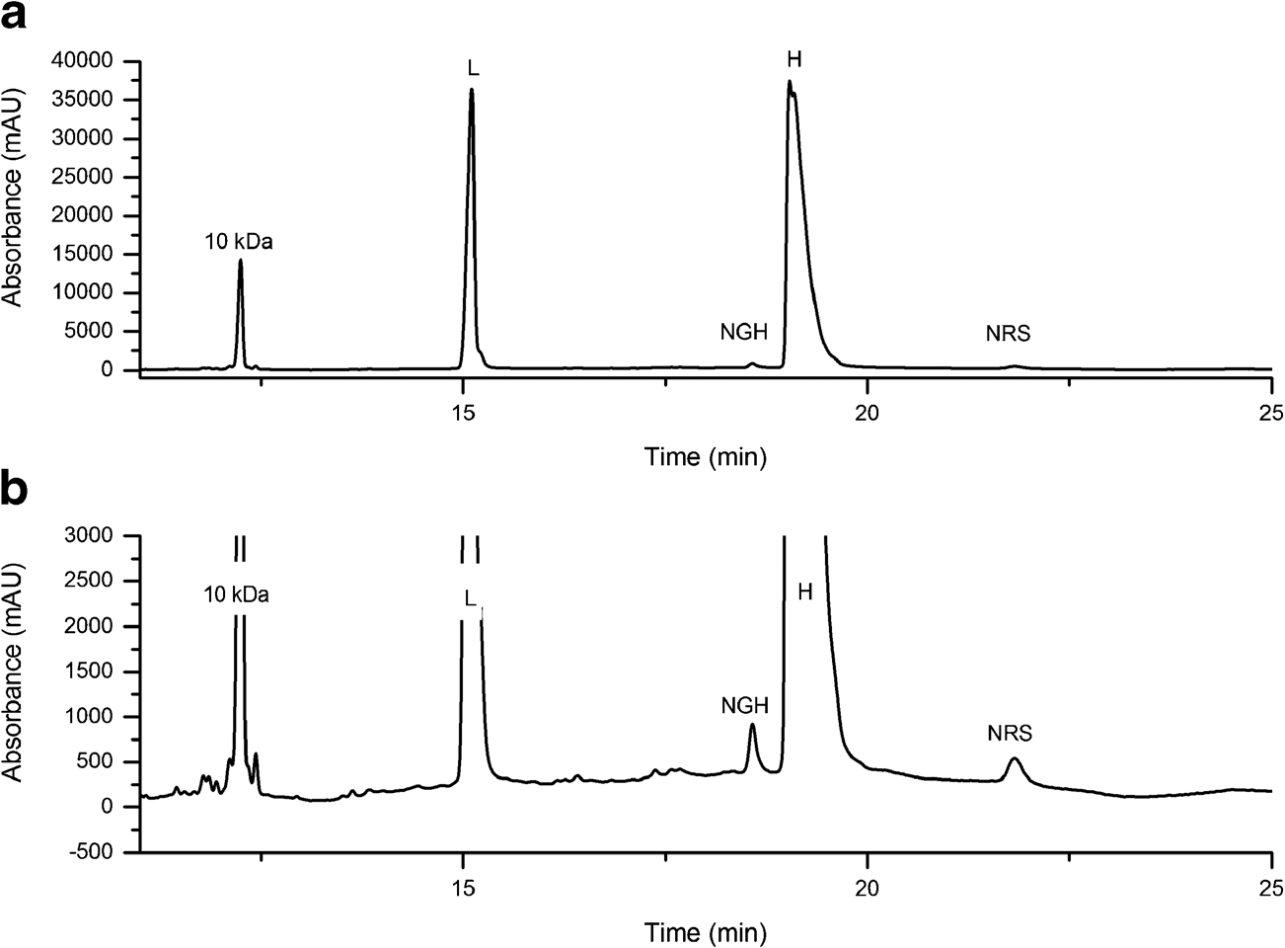
**a** Representative electropherogram of PS 8670 by CE-SDS under reducing conditions. **b** Trace in (a) zoomed in along the Y-axis to show peak shape of NGH and NRS peaks. L = light chain; H = heavy chain; NGH = aglycosylated/non-glycosylated heavy chain; NRS = non-reducible species

A CE-SDS method was developed for quantifying LMW variants in the NISTmAb. The sample is left non-reduced and is alkylated prior to analysis for quantifying mAb fragments, e.g. partial mAbs lacking one or both heavy or light chains. The sample may also be reduced prior to analysis in order to quantify heavy and light chains, heavy chain glycan occupancy, and to monitor the content of non-reducible size variants. A representative electropherogram showing non-reduced PS 8670 analyzed by the optimized CE-SDS assay is given in Fig. 1. Figure 2a and b show reduced PS 8670 analyzed by the optimized CE-SDS assay. Putative peak assignments to LMW combinations of heavy (H) and light (L) chains are based on their apparent molecular weight and migration relative to the monomer, but their exact identity have not yet been evaluated by orthogonal techniques. In the reduced sample, a non-reducible species (“NRS”) was observed with migration time consistent with the monomer. The CE-SDS method was optimized for routine quantification of NISTmAb monomeric purity (reported as the fraction of intact monomer in the non-reduced sample). The same analytical method was also used for evaluating heavy and light chain relative abundance, heavy chain glycan occupancy and non-reducible LMW variant content in the reduced sample.

#### CE-SDS method development

Sample preparation in CE-SDS involves denaturation of the mAb at elevated temperature concomitant with complexation to SDS and, either alkylation of free thiols with iodoacetamide (IAM) or reduction of disulfides with excess beta-mercaptoethanol. Sample preparation optimization is critical for accurate measurement of purity of the non-reduced sample because mAbs are prone to fragmentation during this step, usually due to temperature- or pH-dependent disulfide scrambling and pH-dependent beta-elimination [12, 34]. The separation component of the method is taken from a commercial kit which has been developed and optimized over many years; no further optimization of the separation or detection was deemed necessary for our purposes.

#### Sample preparation optimization

**Fig. 3:**
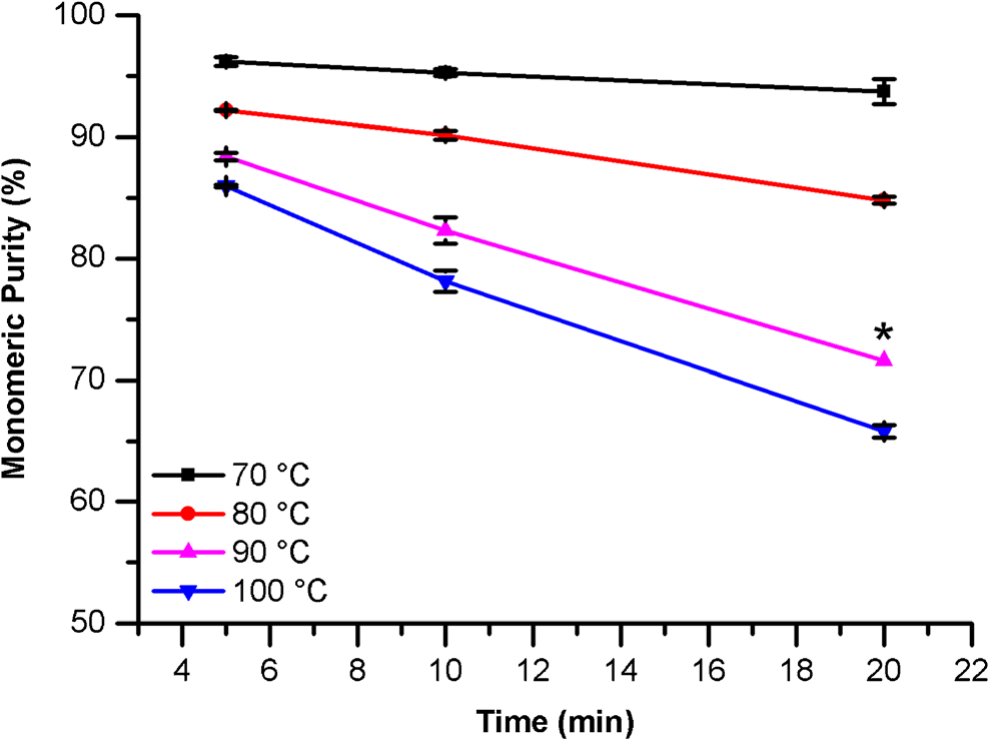
Effect of incubation time and temperature on PS 8670 monomeric purity measured by CE-SDS under non-reducing conditions. Error bars represent 1SD (*n* = 2; ^*^n = 1)

The effect of denaturation time and temperature, SDS sample buffer pH, concentration of alkylating agent (IAM), and time in the instrument autosampler on the measured monomeric purity of the non-reduced sample were evaluated. Samples of PS 8670 were prepared in SDS sample buffer as described, then incubated for the indicated times at a range of temperatures (Fig. 3). The NISTmAb was found to be susceptible to fragmentation during the incubation step, with the level of fragmentation increasing as both incubation time and temperature were increased. Therefore, the gentlest conditions (5 min at 70 °C) were chosen; under these conditions, the peak shape of the monomer is consistent with more stringent denaturation conditions indicating complete denaturation. These denaturation conditions were carried over to preparation of the reduced sample except that the incubation time was increased to 10 min to ensure complete reduction of all disulfide bonds.

**Fig. 4:**
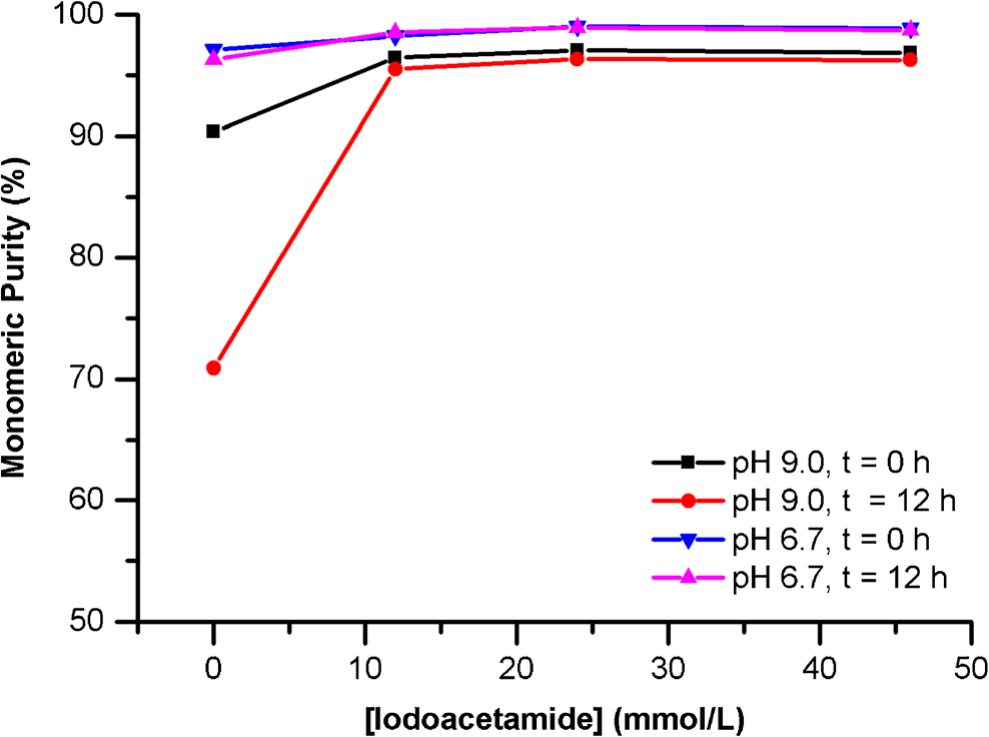
Effect of SDS sample buffer composition and additives on monomeric purity of PS 8670 measured by CE-SDS under non-reducing conditions. Time is approximate time elapsed between replicate injections of the same sample (*n* = 1)

The stability of the protein was compared at pH 6.7 and pH 9.0 in the presence of various concentrations of IAM (Fig. 4). In general, PS 8670 stability is significantly higher in citrate phosphate/SDS (pH 6.7) sample buffer This is particularly evident in the absence of IAM, where the measured monomeric purity of PS 8670 in Tris/SDS pH 9.0 buffer is reduced compared to pH 6.7 and decreases sharply during the time between replicate injections (≈12 h). Addition of IAM greatly improves PS 8670 stability under all conditions and mitigates on-instrument degradation. Based on these data, the optimal SDS sample buffer conditions were determined to be 0.04 mol/L citrate-phosphate/1% SDS sample buffer (pH 6.7) supplemented with 10 μL of 500 mmol/L IAM.

#### Identity of non-reducible species

**Fig. 5:**
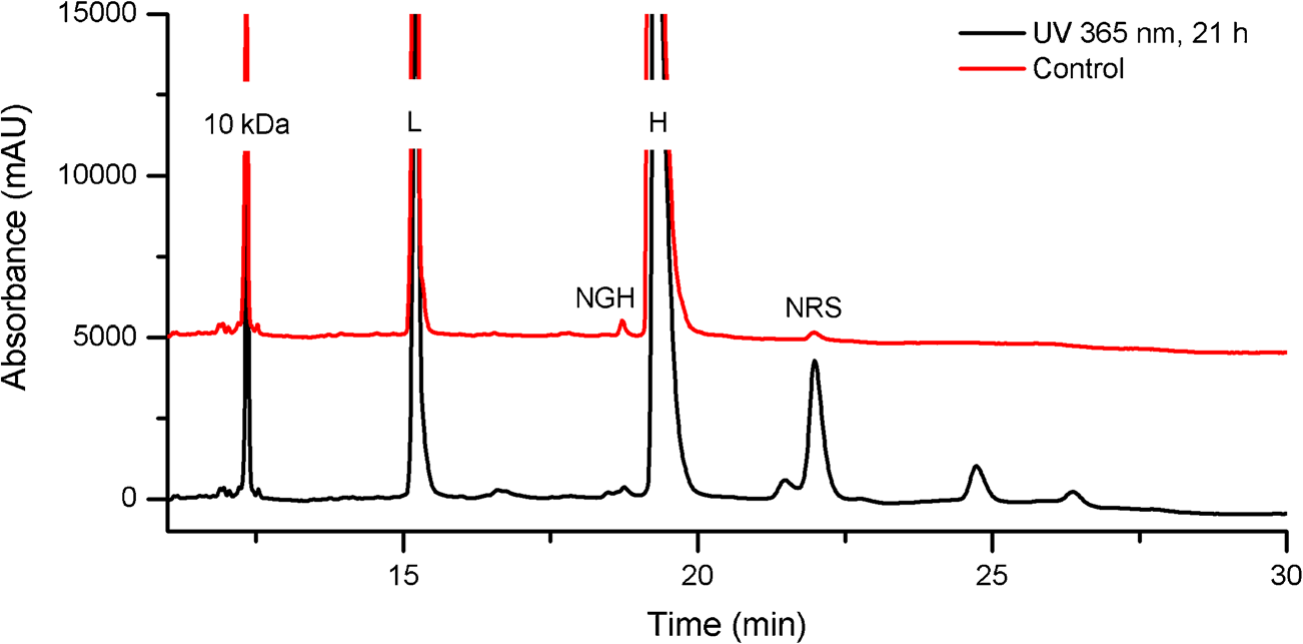
CE-SDS of reduced PS 8670 after UV irradiation. The assay resolves degradation products generated by prolonged exposure to UV radiation, including a peak that co-migrates with the putative H:L thioether (NRS). L = light chain; H = heavy chain; NGH = aglycosylated/non-glycosylated heavy chain; NRS = non-reducible species

In the reduced sample, a small peak is observed which migrates later than the glycosylated heavy chain (Fig. 2). The peak migration time corresponds to a half-antibody fragment consisting of one heavy and one light chain (H:L). A similar peak has been previously observed in another IgG and was attributed to a non-reducible thioether linkage between a heavy and a light chain [35]. Prolonged UV irradiation of PS 8670 leads to an increase in this peak (Fig. 5); UV-induced thioether formation in mAbs has been previously documented [36]. Therefore, we surmise that the non-reducible species co-migrating with the H:L fragment of the NISTmAb is attributable to a H:L thioether linkage.

#### Qualification

The optimized CE-SDS method was qualified for routine purity monitoring of the NISTmAb as part of the Lifecycle Management Plan. The method qualification plan is based on ICH guidelines and is described in detail in [33]. Additional method-specific details can also be found in the ESM.

#### Method linearity

The linearity of the assay response was evaluated for the four peaks detected in the reduced sample (light chain, heavy chain, aglycosylated heavy chain, and H:L thioether). The plots of mean corrected area versus total loading concentration are given in Fig. S2 in the ESM; however, linear regression analysis was performed using each individual data point to allow appropriate statistical fit evaluation. The assay gives an acceptable linear response for the four peaks in the reducing sample from 0.25 mg/mL to 2.0 mg/mL total loading concentration. R-squared values ranged from 0.999 for the heavy chain (2.3% relative residual standard deviation) to 0.939 for the thioether species (17.6% relative residual standard deviation); see Table S2 in the ESM. The higher relative residual standard deviation of the thioether and aglycosylated heavy chain linear fit is attributed to the difficulty of reproducibly integrating very low abundance species; however, adequacy of the linear fit to this curve is confirmed with an F test as indicated in ESM Table S2. The linear range of the assay corresponds to 25% to 200% of the target concentration of the reduced sample (1.0 mg/mL).

#### Limit of detection/limit of quantification

The limits of detection and quantification (LOD and LOQ, respectively) of the method were estimated from the signal-to-noise ratio (SNR) value of the aglycosylated heavy chain minor variant (NGH) in the reduced sample at the target loading concentration (1.0 mg/mL). The LOD and LOQ in units of mass were 16 (2.5) pg (standard deviation, SD) and 53 (8.4) pg, respectively. The mass-based values were converted to % purity units and the LOD and LOQ of the method at the target loading concentration were determined to be 0.17 (0.03) % relative abundance (RA) and 0.57 (0.09) % RA, respectively. See the ESM for relevant calculations.

#### Specificity

Method specificity with respect to potential matrix interferents was assessed by documenting the absence of interfering species in blank injections prepared with and without the 10 kDa internal standard. Blank samples were prepared identically to true samples and analyzed by CE-SDS under reducing and non-reducing conditions. No interfering peaks were observed attributable to the formulation buffer. In the case of IAM treatment, a reagent peak was observed that did not co-migrate with PS 8670 peaks. Electropherograms of blank samples containing the internal standard contained only the internal standard main peak and its low-level impurities, none of which co-migrated with known peaks in PS 8670. The total carryover of the method was confirmed to be negligible based on no detectible signal (other than IAM reagent peak) for a blank injection immediately following a sample injection at the end of each sequence. Specificity with respect to potential degradants was evaluated using forced degraded PS 8670 which had been subjected to UV light for 21 h prepared as described in the ESM. Photo-induced intra-chain fragmentation and other degradation products were observed as new peaks that did not co-migrate with known unstressed PS 8670 peaks (Fig. 5).

#### Precision

Intermediate precision of the method under non-reducing and under reducing conditions was estimated from 12 injections of PS 8670 over two capillaries and four days as in described in ESM Table S3 (four days for each sample, reduced or non-reduced, total of eight days). The parameters recorded for each electropherogram and associated calculations are detailed in the ESM. The resultant intermediate precision values are given in Table 1, each of which resulted in a coefficient of variation (CV) < 5%, and were determined to be a suitable for monitoring NISTmAb quality over its lifecycle.

**Table 1:** Intermediate precision of optimized CE-SDS assay

Parameter	Mean ± *u*_*c*_^a^	CV
Instrument Qualification (IQ) Standard		
10 kDa internal standard migration time (min)	12.45 ± 0.12	1.0%
100 kDa marker migration time (min)	21.97 ± 0.21	1.0%
PS 8670		
Monomer migration time (min)^b^	28.19 ± 0.28	1.0%
Monomeric purity (%)^b^	98.79 ± 0.38	0.4%
Light chain migration time (min)^c^	15.29 ± 0.18	1.2%
Heavy chain migration time (min) ^c^	19.29 ± 0.23	1.2%
Light chain relative abundance (%)	32.02 ± 0.20	0.6%
Heavy chain relative abundance (%)	67.26 ± 0.20	0.3%
Glycan occupancy (%)^c^	99.40 ± 0.01	0.01%
NRS relative abundance (%)^c^	0.31 ± 0.02	4.9%

^a^Stated uncertainty represents the intermediate precision reported as a combined standard uncertainty, at a level of one standard deviation, based on ANOVA analysis as described in ESM(*n* = 12)

^b^non-reduced sample

^c^reduced sample

#### Performance criteria

The IQ standard and PS 8670 were used to evaluate system performance and system suitability, respectively, during NISTmAb RM 8671 Value Assignment [29]. The performance criteria for the method were set for each parameter based on the measured intermediate precision. These criteria are useful for ensuring that the analytical method is in control, thus establishing confidence in the data acquired using the method. Performance criteria for the optimized CE-SDS method can be found in ESM.

### Monitoring NISTmAb size variants with SEC

**Fig. 6:**
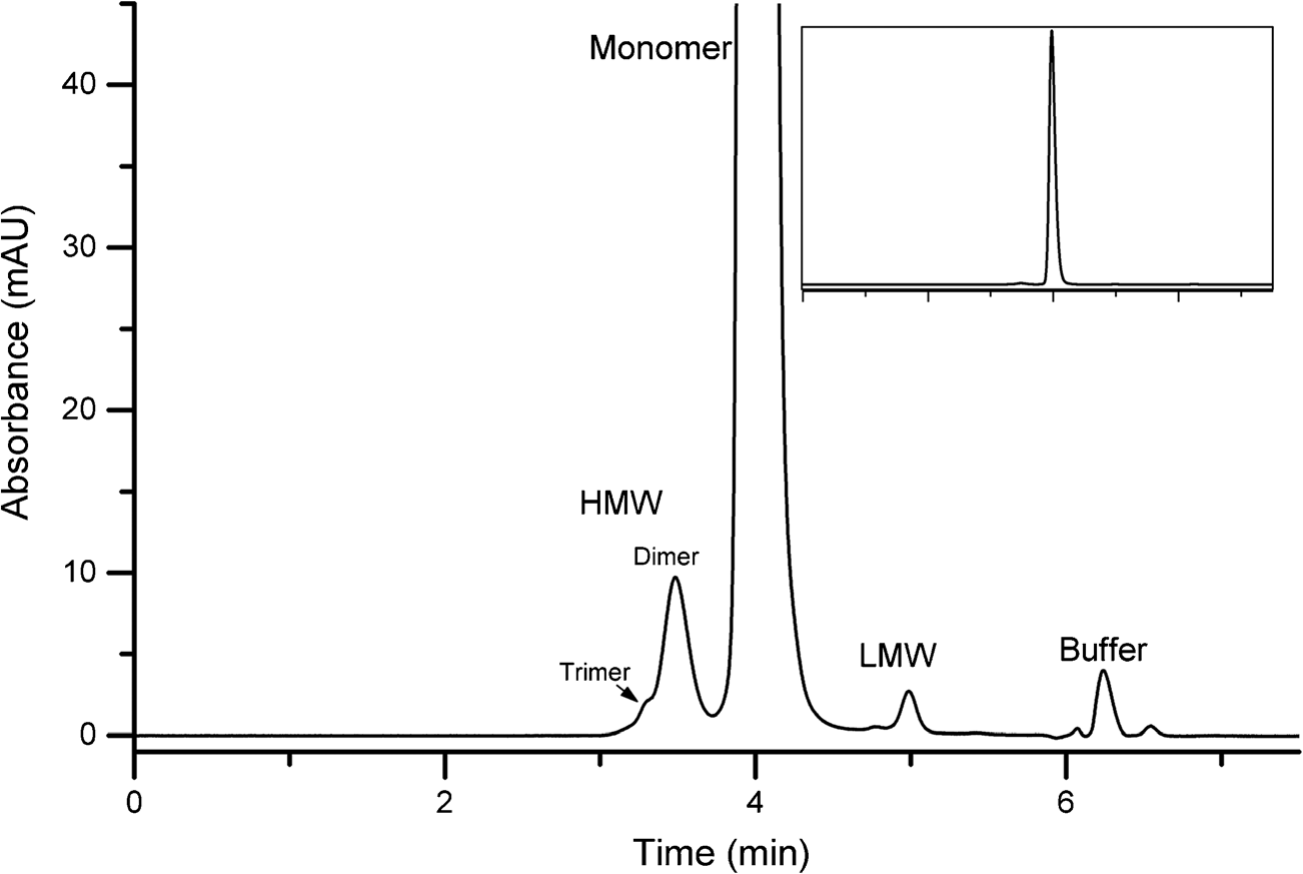
Representative SEC chromatogram of PS 8670 zoomed in the Y-axis; inset shows expanded chromatogram. HMW = high molecular weight (trimer and dimer), LMW = low molecular weight. Peak labeled buffer is void volume of the column from L-histidine background buffer

An SEC method was also developed for monitoring monomeric purity in the NISTmAb. The method was optimized using a design of experiments central composite design (CCD) approach. A representative chromatogram showing PS 8670 analyzed by the optimized SEC method (mobile phase consisting of 100 mmol/L sodium phosphate 250 mmol/L sodium chloride buffer, pH 6.8) is given in Fig. 6. Peaks were identified as either high or low molecular weight species based on their migration time in relation to the monomer. In the HMW series there is evidence of both trimer and dimer peaks. In the LMW species there is evidence of two resolved fragment peaks that elute immediately after the monomer peak. Note that the peak at approximately 6.25 min was verified to be the void volume of the column and due to elution of the L-histidine sample background buffer. Details on the SEC optimization for routine quantification of NIST mAb monomeric purity is discussed in more detail below.

#### SEC method development

The PS 8670 was initially received in 2012 and a small batch of material was distributed to collaborators for analysis as part of the ACS book series “State-of-the-art and emerging technologies for therapeutic monoclonal antibody characterization” [5–7]. As part of this series, a chapter on separation science methods for the analysis of monoclonal antibodies was prepared, which included the size exclusion chromatography method selected here for further evaluation [8]. The original method was applied at a flow rate of 0.4 mL/min. It is well known based on the van Deemter equation that a partial reduction in mobile phase linear velocity (i.e. lower flow rate) will result in a more efficient separation [37]. It was therefore determined that further optimization be performed using a flow rate of 0.3 mL/min because the UHPLC method would still allow for sufficiently high throughput.

Non-specific adsorption to the SEC stationary phase (i.e. to exposed silanol groups) may result in peak broadening and loss in resolution. These ionic interactions, which constitute a non-size-exclusion contribution to the SEC separation, may be minimized by adjusting the mobile phase pH in relation to protein pI, addition of organic modifiers, and/or an increase in ionic strength to shield the protein/support interaction [38–40]. A CCD design was constructed to evaluate the relative influence of Na_3_PO_4_ and NaCl concentration (and thus ionic strength) on method performance [41]. ESM Table S12 shows the calculated buffer concentration values used in the CCD experiment. PS 8670 (6 μL of 10 μg/μL NISTmAb) was injected at a flow rate of 0.300 mL/min for 10 min at ambient temperature using each of the indicated buffers. The control injections were run at the beginning of each day and the values were an average of 12 injections over 4 days; individual buffer concentration values were the average of 3 injections.

**Fig. 7:**
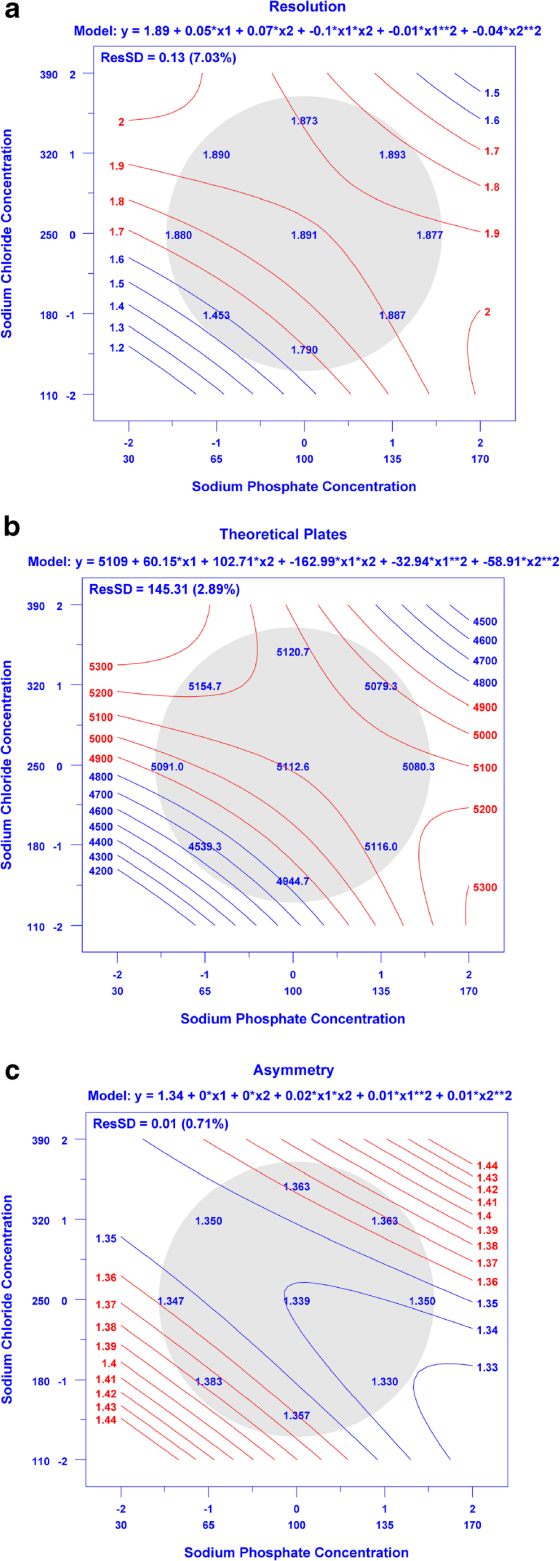
**a-c** Contour plots showing effect of sodium phosphate and sodium chloride concentration on (**a**) resolution, (**b**) monomer efficiency, and (**c**) asymmetry. Values listed in the shaded region represent mean performance metrics for the (0,0) control point (*n* = 12, 3 injections on each of four days), for all other points *n* = 3 on one day. Contour lines represent a quadratic fit to the data using Dataplot

The resultant chromatograms were integrated according to the integration parameters in ESM Table S17 to evaluate effects of buffer condition on resolution (dimer and monomer, *R*_*s*_), number of theoretical plates (monomer, *N*), and monomer peak asymmetry (*A*_*s*_). The mean method performance at the control points was determined to yield *R*_*s*_ = 1.89 (0.04), *N* = 5112 (142), and *A*_*s*_ = 1.34 (0.02) (SD). Each of the measured CCD data points resulted in values of *R*_*s*_, *N*, and *A*_*s*_ (Fig. 7 a, b, and c respectively) within 3SD of the control point other than the data points in the lower left hand region of the response surface. Visual inspection of the chromatograms also demonstrated that the separation of HMW, monomer, and LMW species was affected at the lowest NaCl and NaPO_4_ concentration. Dataplot software was used to make contour plots using a quadratic model that relates each figure of merit to sodium phosphate and sodium chloride concentration in the buffer solution (Fig. 7 a-c) [42, 43]. The quadratic model indicates movement towards lower NaCl and/or NaPO_4_ concentrations would reduce method performance according to these metrics, with all other points yielding similar or only moderate improvement in performance. The minor advantage that may be achieved in going to higher NaCl and/or NaPO_4_ is outweighed by the potential for additional instrument wear and difficulty in degassing the higher ionic strength solvents. Ultimately the control point method (100 mmol/L NaPO_4_ and 250 mmol/L NaCl, pH 6.8) was chosen as the final method as it provided resolution, efficiency, and peak asymmetry at or near the optimum as observed in the CCD. Additional details of the CCD can be found in the ESM.

#### Qualification

The optimized SEC method was qualified for routine purity monitoring of the NISTmAb as part of the Lifecycle Management Plan. The method qualification plan is based on ICH guidelines and is described in detail in [33]. Additional method-specific details can also be found in the ESM.

#### Method linearity

The linearity of the assay response was evaluated for the four peaks detected (trimer, dimer, monomer, and fragment). The plots of mean area versus loading concentration are given in Fig. S4 in the ESM section; however linear regression analysis was performed using each individual data point to allow appropriate statistical fit evaluation. The assay shows acceptable linearity of response for all peaks from 18 μg to 102 μg total protein load. The R-squared values ranged from 0.999 for the monomer peak (0.6% relative residual standard deviation) to 0.991 for the trimer peak (4.5% relative residual standard deviation); see Table S14 in the ESM. The linear range of the assay corresponds to a range of 30% to 170% of the target total protein load of 60 μg.

#### Limit of detection/limit of quantification

The limits of detection and quantification (LOD and LOQ, respectively) of the method were estimated from the signal-to-noise ratio (SNR) value of the fragment peak (LMW species) at 30% of the target load as described in the ESM. The LOD/LOQ in units of mass were 3.71 (0.25) ng (SD) and 12.39 (0.83) ng, respectively. The mass-based values were converted to % purity units at the target load (60 μg total protein injected) as described in the ESM. The LOD and LOQ of the method at the target load were determined to be 0.006 (0.0004) % RA and 0.021 (0.001) % RA, respectively.

#### Specificity

The specificity of the assay with respect to potential matrix interference was assessed by verifying the absence of interfering peaks in the blank and demonstrating negligible carryover. Blank samples consisting of PS 8670 formulation buffer were analyzed using the optimized method parameters. Only one peak was present in the blank, attributed to the histidine component of the buffer, and there was no significant interference at the elution time of any of the peak groups as given in Fig. 6. The % carryover was assessed by running a blank-target concentration-blank sequence with the target sample load being 60 μg. The total carryover of the method was confirmed to be negligible based on no detectible signal (other than L-His reagent peak) for a blank injection immediately following a sample injection.

**Fig. 8:**
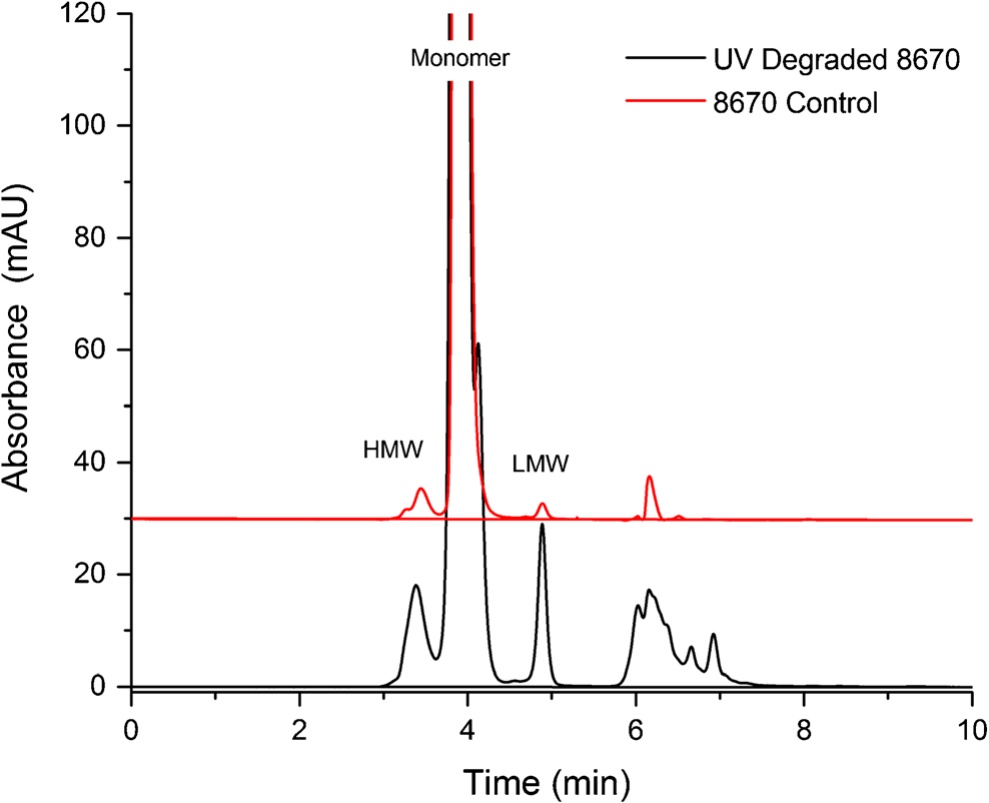
SEC of UV-treated PS 8670. The assay resolves degradation products generated by prolonged exposure to UV radiation, including a new species observed as a shoulder peak on the monomeric peak. HMW = high molecular weight, LMW = low molecular weight

Percent recovery based on absorbance at 280 nm was also calculated as described in the ESM to further demonstrate specificity and infer accuracy. The method uses the on-column peak area and theoretical NISTmAb extinction coefficient to estimate protein mass eluting from the column, and was applied to all linearity injections. This method of percent recovery determination demonstrated the effective elution of >99% (103.73 (0.521)) of the protein from the column as seen in Table S15 in the ESM. Specificity with respect to potential degradants was evaluated using forced degraded material. The assay was applied to analysis of PS 8670 which had been subjected to UV light for an extended period. Both increased aggregation (HMW species) as well as increased fragmentation (LMW species) was observed. In addition, a new species was observed following UV degradation as a peak shoulder on the monomeric peak (Fig. 8).

#### Precision

The intermediate precision of the method was estimated from 24 injections of PS 8670 over four columns and 8 days (2 days per column) as seen in ESM Table S16. The parameters recorded for each chromatogram and associated calculations are detailed in the ESM. The intermediate precision values are given in Table 2, each of which resulted in a CV less than 5% with the exception of the HMW relative abundance (≈11%). The slightly larger CV for the HMW species is likely due to incomplete resolution of contributing species, and ultimately was determined to be suitable for monitoring NISTmAb aggregation.

**Table 2:** Intermediate precision of optimized SEC

Parameter	Mean ± *u*_*c*_^a^	CV
Instrument Qualification (IQ) Standard		
γ-globulin Migration Time (min)	3.90 ± 0.08	2.0%
Ovalbumin Migration Time (min)	4.69 ± 0.10	2.0%
PS 8670		
Monomeric Purity (%)	98.78 ± 0.12	0.1%
HMW Relative Abundance (%)	1.02 ± 0.12	11.6%
LMW Relative Abundance (%)	0.20 ± 0.01	4.1. %
Monomer Retention Time (min)	3.95 ± 0.08	2.1%
Resolution (Dimer-Monomer)	1.95 ± 0.05	2.7. %

^a^Stated uncertainty represents the intermediate precision reported as a combined standard uncertainty, at a level of one standard deviation, based on ANOVA analysis as described in ESM (*n* = 24)

#### Performance criteria

The IQ standard and PS 8670 were used to evaluate system performance and system suitability, respectively, during NISTmAb RM 8671 Value Assignment [29]. The performance criteria for the method were set for each parameter based on the measured intermediate precision. These criteria are useful for ensuring that the analytical method is in control, thus establishing confidence in the data acquired using the method. Performance criteria for the optimized SEC method can be found in ESM.

#### Accuracy and method comparison

In the case of qualified physicochemical assays described herein, the intent is to provide method-specific reference quantity values in the form of relative charge or size purity values (as opposed to SI traceable values) to be used as method performance criteria. Accuracy is therefore inferred from analytical method figures of merit (precision, linearity, and specificity) as described in the sections above. Accuracy can be further evaluated based on comparison of the related, yet orthogonal techniques applied herein, with a consideration of the method-specific nuances.

Comparison of monomeric purity by SEC and CE-SDS of the non-reduced sample (Table 3) reveals excellent agreement between the orthogonal qualified assays; the relative proportion of monomer is the same when measured by either method. Apparent differences are observed in the HMW and LMW species; however, this is not surprising considering the orthogonality of these approaches. SEC is run herein under non-denaturing conditions, whereas CE-SDS denatures non-covalent interaction prior to analysis. SEC was therefore able to detect HMW variants (dimer and trimer) which were not detectable by CE-SDS with UV detection. The absence of detectable HMW species in CE-SDS implies that any covalent aggregates present are at very low levels; this is confirmed by other work using fluorophore-labeled PS 8670 and CE-SDS with fluorescence detection, where low levels of HMW (≈0.1% RA) species were detected [44]. Taken together, these data suggest that the PS 8670 contains low levels of aggregates which dissociate under denaturing conditions (e.g. non-covalent oligomers).

**Table 3:** Comparison of PS 8670 size variants by method

Non-Reduced Sample	Monomer (%)	LMW Variants (%)	HMW Variants	
SEC-UHPLC (qualified)^a^	98.8 ± 0.1	0.2 ± 0.01	1.0 ± 0.1	
CE-SDS-UV (qualified)^a^	98.8 ± 0.4	1.2 ± 0.4	ND	
Reduced Sample	Light Chain (%)	Heavy Chain (%)	Non-Reducible Species (%)	Heavy Chain Glycosylation (%)
CE-SDS-UV (qualified)^a^	32.0 ± 0.2	67.3 ± 0.2	0.3 ± 0.01	99.4 ± 0.01

*ND* not detected; *UHPLC* ultrahigh pressure liquid chromatography; *UV* UV detector

^a^Stated uncertainty represents the intermediate precision reported as a combined standard uncertainty, at a level of one standard deviation, based on ANOVA analysis as described in ESM

Similarly, the relative abundance of LMW species measured by SEC is lower than that measured by CE-SDS of the non-reduced sample. This may be due to differences in resolution of LMW variants by the two methods, release and observance of fragments in CE-SDS that were originally held in place by non-covalent interactions, or the presence of some low level of induced fragmentation that is unavoidable in CE-SDS due to sample preparation. Regardless, the LMW variants are within ±3*u*_*c*_ of the mean, indicating good agreement between the methods.

CE-SDS of the reduced sample provides complementary information to the measures of monomeric purity obtained by SEC and CE-SDS of the non-reduced sample. Reduction of inter-chain disulfide bonds within the monomer and LMW variants allows their composition to be studied in greater detail. The identity of the fragment species in the non-reduced sample consisting of disulfide-linked H and L chain combinations (Fig. 1) is confirmed by their reduction to the corresponding H and L chain components (Fig. 2), resulting in an H + L chain relative abundance of ≈99.3%. This value is higher than the measured monomeric purity of the non-reduced sample by CE-SDS or SEC, which confirms that the majority of the size variants detected by these two methods are either disulfide-linked or noncovalent in nature. Size variants detected in the reduced sample represent covalent, non-reducible modifications such as the non-reducible inter-chain linkages (thioether) or intra-chain fragmentation (not detected above LOD in the current assay). Lastly, CE-SDS of the reduced sample provides excellent resolution of the glycosylated and aglycosylated heavy chain peaks allowing quantification of glycan occupancy of the heavy chain (Fig. 2).

The resultant values and associated uncertainties for PS 8670 can be used as a basis for comparison with values of the same kind for physicochemical attributes of the NISTmAb. The size heterogeneity metrics measured by the qualified methods were therefore also compared to historical data from the previous crowdsourced analysis (Table 4) [8]. Excellent agreement was observed between the results from the qualified SEC and CE-SDS of the non-reduced samples; all values reside within ±3*u*_*c*_ associated with the qualified value.

**Table 4:** PS 8670 size variants by method from ACS book series [8]

Non-Reduced Sample	Monomer (%)	LMW Variants (%)	HMW Variants	
SEC-UHPLC (book)	98.7	0.3	1.0	
CE-SDS-UV (book)	97.8	2.2	ND	
Reduced Sample	Light Chain (%)	Heavy Chain (%)	Non-Reducible Species (%)	Heavy Chain Glycosylation (%)
CE-SDS-UV (book)	32.3	66.1	0.4	99.3

*ND* not detected; *UV* UV detector

The measured relative abundances of light chain, heavy chain, and non-reducible species in the reduced sample by the qualified CE-SDS method were also compared to historical data [8]. Good agreement between qualified and historical values was observed in all cases, although minor differences in heavy chain and non-reducible species relative abundance and in heavy chain glycan occupancy were observed. The historical crowdsourcing data was collected as a characterization effort, and thus replicates were not run to allow full statistical comparison; however, it is likely that the differences are attributable to integration parameters and/or sample handling/preparation [8].

The qualified values for all methods reported here should not be taken as absolute; different values may result from changes in sample preparation, analytical method, data analysis, and/or integration parameters. The qualified values reported here are, however, fit for their intended purpose which is to provide expected method performance for PS 8670. PS 8670 and the method-specific performance criteria will therefore be used as a system suitability control during RM 8671 value assignment as described in [29].

### Monitoring NISTmAb size variants with dynamic light scattering

Dynamic light scattering (DLS) analysis was performed to analyze larger size variants of the NISTmAb ranging in size from a few nanometers to approximately 1 μm. This technique was used primarily to obtain informational values and to assess the stability of a subset of the vialed PS 8670 and RM 8671 material [29]. DLS is not a very quantitative technique therefore quantification of the monomeric or higher organized species content is not possible as with the other methods described above. The method development and optimization procedures for DLS, therefore, were not as rigorous as for the SEC or CE-SDS methods. This technique is mostly used qualitatively, to monitor the presence of monomeric and larger species as a function of different conditions. The method employed for analysis of PS 8670 and RM 8671 has been described in the ESM section; further method optimization was not deemed necessary for our purposes.

#### Repeatability, intermediate precision, and sizing accuracy

Solutions of nominally 200 nm diameter polystyrene microspheres were assessed over a span of four days to measure the sizing accuracy and intermediate precision of the instrument. Over the span of four days, the mean hydrodynamic diameter of the microspheres was measured to be 208 (2) nm (SD). The intra-day measurements for microspheres ranged from 206 nm to 209 nm. These measurements were within the acceptable range as allowed by the instrument manufacturer.

**Fig. 9:**
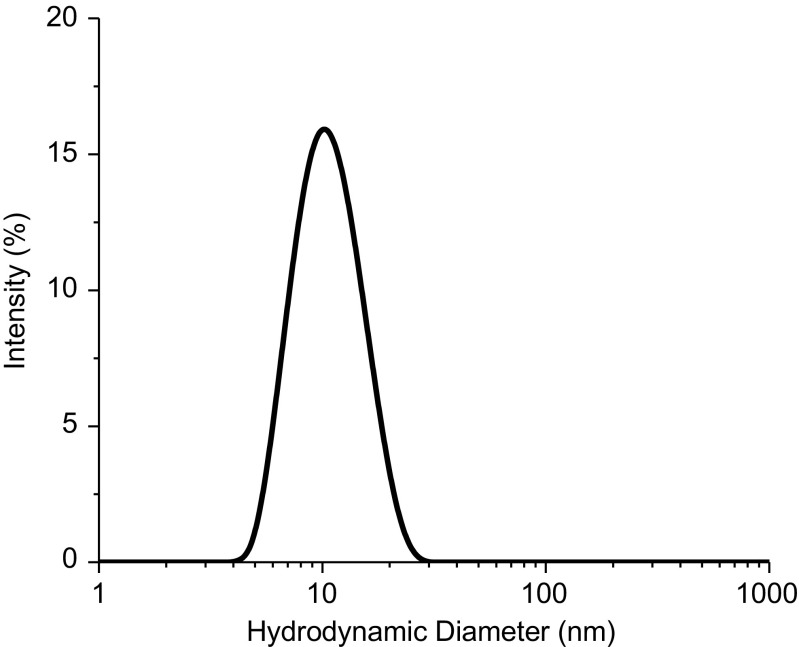
Representative intensity based plot of PS 8670

Subsequently, 8 separate vials of the PS 8670 material were measured in triplicates over a span of 3 days to assess inter-day, or intermediate precision, and intra-day variability in the sizing results. The size distribution obtained is a plot of the relative intensity of light scattered by particles of various sizes and is called the intensity size distribution. The peak, corresponding to the monomeric form of the protein, is displayed in the representative intensity plot for PS 8670 in Fig. 9. The main peak is located at 10 nm, which is characteristic of the monomer peak. There seems to be a slight increase in the baseline around 1000 nm as well, indicating the potential presence of larger particles. However, since 1000 nm is towards the higher end of detection capability of the instrument, the sizing results above 1000 nm are generally not reliable. Results under intermediate precision conditions of the hydrodynamic diameter of the protein was 10.2 (0.2) nm (SD) with a CV of 1.8%, while results under the intra-day conditions ranged from 10.1 nm to 10.3 nm, with the CV ranging from 1.1% to 2.3%. The repeatability and intermediate precision values for the microspheres and the PS 8670 indicate DLS’s inherent variability in sizing spherical nanoparticles and irregular, translucent particles in protein solutions.

Studies performed above were to understand the repeatability, sizing accuracy, and precision of the instrument using microspheres and PS 8670. Understanding this precision is useful for ensuring that the analytical method is in control, thus establishing confidence in the data acquired using the method. From these measurements, it was determined that the technique was suitable for its purpose to monitor NISTmAb aggregation.

### Monitoring NISTmAb size variants with flow imaging

Flow imaging analysis was performed to analyze larger size submicrometer aggregates of the NISTmAb. Like DLS, this technique was primarily used to obtain informational values and to assess the stability of a subset of the vialed PS 8670 and RM 8671 material [29]. Method development and optimization studies to understand the repeatability, intermediate precision, and sizing accuracy were performed to better understand the capabilities of the technique and to allow comparison of different data sets; a full qualification was not performed.

#### Method development and optimization

The effect of various instrumental parameters, method variations, and sample handling on observed particle concentrations using different sample types were assessed to determine the optimum method. The effect of degassing, purge and prime volumes, different solutions on the optimization step, and sample handling were investigated using different sample types. For these studies, where large volumes of aggregated protein solution were required, a commercially available polyclonal IgG solution was intentionally aggregated, as described in the ESM section, and then measured. Protein particles particle concentration is reported for one or more size bins as an equivalent circular diameter (ECD), which is defined as the diameter of a polystyrene microsphere with the same image area as the observed particle. The effect of method variation on subvisible particle concentrations in the polyclonal IgG is shown in Table S18 in the ESM. It was determined that the effect of degassing was minimal, while samples analyzed using a prime volume of 2 mL displayed lower variability in the concentrations compared to the other prime values studied. Additionally, using a purge volume of 200 μL and optimizing with water seemed most effective and convenient. Sample handling may lead to slight variations in FI results. To quantify if such a variability exists, two analysts analyzed the polyclonal IgG sample by slightly modifying their mixing procedures; they either tilted or swirled the vial prior to dispensing the sample. Table S19 (see ESM) shows that the variability in the concentrations measured between analysts is relatively small among all the size ranges.

Proteins tend to adsorb to a multitude of interfaces, including tubing and glass surfaces within an instrument, which would greatly affect the particle concentrations obtained for each run, especially if the protein was being desorbed during subsequent runs. Therefore, to check if PS 8670 material adsorbs onto the flow cell and tubing, a series of different solutions (histidine buffer with and without a small percentage of Tween 20 and PS 8670) were run in a specific order. Table S20 (see ESM) shows the resulting particle concentrations as various solutions are passed through the instrument. The order of runs is very important as it can identify protein adsorption problems. From ESM Table S20, there were only minor increases in subvisible particle concentrations in the buffers after the PS 8670 material has been run, indicating adsorption of the protein is not a problem. See ESM for details regarding method development and optimization experiments and for more detailed results.

#### Repeatability, intermediate precision, sizing accuracy, and counting accuracy

To assess the repeatability, count accuracy, size accuracy, and precision associated with this technique, a series of experiments were performed using nominally 5 μm commercial count-standard (Count-Cal) microspheres, 2 μm polystyrene, and 10 μm polystyrene microspheres. Additionally, a new protein-like surrogate made of ethylene tetrafluoroethylene (ETFE) in solution was analyzed because these particles mimic the optical properties of proteinaceous particles, but lack the corresponding stability problems. More details regarding the experiments and the results are discussed in the ESM section.

Initially, the Count-Cal concentration measurements were higher than the manufacturer’s specifications so a primary bead standard was used to obtain a concentration correction factor [45]. This correction factor was calculated by taking the ratio of the known concentration of the calibrated beads in the primary bead standard versus the measured concentration of the same beads on the FI instrument. The correction factor was then applied to all of the raw concentration data throughout this document to adjust for the larger-than-expected thickness of the flow-cell [45]. Over the span of 5 days, the Count-Cal solution, corrected for FI cell thickness, showed little variability in concentration, with CV of 3%, and in size, with CV of 2% (ESM Table S21). Table S21 shows that the variabilities in concentration and size are within the acceptable range of the instrument (concentration repeatability is ±5%, and sizing repeatability ±5%, according to the instrument manual). The 2 μm and 10 μm microspheres were also run to study the repeatability in the concentration and size measurements. The concentration measured for both bead sizes was reproducible over three runs, with CVs ≤ 2%.

Irregular shaped ETFE particles, run over three days, were assessed for particle concentrations. Variabilities (inter-day and intra-day) in concentrations were minimal, as shown in Table S21 in the ESM. For all size bins analyzed, the CVs for intermediate, or inter-day, precision were ≤6%. While these CV values are higher than for the microspheres, they still represent high repeatability for irregular shaped particles. This data suggests that even non-spherical particles, as long as they are not sticky and are inert, can be sized and counted reproducibly by this technique.

#### Determination of optimum sampling protocol

Based on the repeatability and intermediate precision values from all experiments, an optimum sampling procedure was developed. In all cases, the CVs obtained were satisfactory and consistent with the instrument specifications*.* From these studies, the following variables were chosen to be used on the PS 8670 and RM 8671 analysis: no degas, prime volume = 2 mL, purge volume = 200 μL, optimization with water. Further sample handling procedures are detailed in the ESM.

#### Measurement of PS 8670 using optimized method

Representative samples of PS 8670 were run using the optimized procedure to obtain protein particle concentrations over the course of 2 days (4 separate vials, 1 run from each vial). All particles with an ECD ≥ 2 μm were reported in a single size bin as the total particle concentration. Table 5 shows the mean subvisible particle concentrations of PS 8670 samples, along with the controls, DIUF water and buffer. The range of protein concentration (minimum to maximum calculated concentration for each sample type) that could be present in the particles is also shown. Eq. S17 and Eq. S18 in the ESM section were used to calculate the range of estimated protein concentration within the total number of particles (ECD ≥ 2 μm). The range represents the span of concentration values; it is obtained by calculating the protein concentration within the particles (Eq. S17) for each run of a particular sample and then simply reporting the minimum and maximum concentration values among the series of those runs. These concentration values are then converted into percentage of protein in the particles relative to the total protein in solution of the unstressed sample. These calculations are only approximations. The range of concentration values is reported to represent the relatively large CV observed in the unstressed NISTmAb samples and to help to establish whether the particle concentrations are physically relevant in terms of the protein concentration bound up in those particles. A more detailed description of these equations are described in Kalonia et al. [46].

**Table 5:** Subvisible particle concentrations (ECD ≥ 2 μm) observed in the controls (DIUF water, buffer, 5 μm Count-Cal) and PS 8670

Samples	*Particle Concentration ECD* ≥ 2 μm (mL^−1^), *n* ≥ 3	CV (%)	Range of Concentration of Protein in Particles (ng/mL)	^a^Range of Protein in Particles/Protein in Solution (%)
DIUF water	12 (5)	–	–	
Buffer	42 (32)			
5 μm Count-Cal	2898 (32)	1		
8670 *n* = 4 (2 days)	4271 (1413)	33	105 to 449	0.0013 to 0.0045

^a^Total protein concentrations from referenced UV absorbance values [29] were used to approximate the ratio of the concentration of protein in the particles to the concentration of protein in solution. The uncertainty is expressed as (SD)

## Conclusion

Size heterogeneity measurement of therapeutic proteins represent a diverse set of tools, driven largely by the need for measurement capabilities spanning four orders of magnitude. The current manuscript describes optimization of a subset of size-based measurements using the NISTmAb primary sample 8670 (PS 8670), followed by method qualification to ensure suitability for their intended purpose. CE-SDS and SEC were optimized as orthogonal technologies to monitor monomeric purity with selectivity for antibody fragmentation and aggregation (dimer, trimer, and tetramer). CE-SDS and SEC method qualification using PS 8670 yielded method-specific performance criteria to be used as a system suitability control during RM 8671 reference value assignment. A dynamic light scattering method was also developed to provide average hydrodynamic radius measurement. Finally, FI was extensively optimized and qualified to ensure appropriate calibration and measurement of sub-micron particulate content. DLS and FI provide orthogonal, rich datasets critical to the quality monitoring the NISTmAb colloidal stability and will be incorporated for informational value assignment of RM 8671. The NISTmAb, along with associated assays described herein, will serve as a common test case through which these orthogonal methods can be compared and refined across many laboratories and analysts to promote comprehensive understanding of the methods and best practices for their use.

## Electronic supplementary material

ESM 1 (PDF 3.52 MB)
