# Supplementary material for: Development of orthogonal NISTmAb size heterogeneity control methods
Source: Anal Bioanal Chem. 2018 Feb 10;410(8):2095–110. doi: 10.1007/s00216-017-0819-3 (PMC5830496; doi:10.1007/s00216-017-0819-3)
Supplement: Supplementary file 1 — (PDF 3.52 MB) [file 216_2017_819_MOESM1_ESM.pdf]

Analytical and Bioanalytical Chemistry

Electronic Supplementary Material

Development of orthogonal NISTmAb size heterogeneity control methods

Abigail Turner, Katharina Yandrofski, Srivalli Telikepalli, Jason King, Alan Heckert, James Filliben, Dean Ripple, John Schiel

CE-SDS Materials and Methods

Buffer Preparation. Mass measurements for solution preparation were performed on a Metler Toledo AL54 balance. Formulation buffer (12.5 mmol/L L-histidine/12.5 mmol/L L-histidine HCl, pH 6.0) was prepared as follows: 1) Weigh out 1.3129 g histidine monohydrochloride monohydrate and 0.9704 g L-histidine and dilute with ≈450 mL type 1 deionized ultrafiltered water (DIUF) that was obtained from an ultrapure water system fed with reverse osmosis water and filtered through a 0.2 µm filter; 2) while recording pH with a calibrated pH meter, adjust pH by drop-wise addition of 1 mol/L hydrochloric acid to 6.00±0.02; 3) transfer to a 500 mL volumetric flask, rinse beaker with DIUF water and adjust flask volume to 500 mL using the rinse water; 4) sterile filter using a 0.22 µm cellulose acetate membrane into a sterile plastic bottle; 5) store at 2 to 8 °C.

Citrate-phosphate/SDS sample buffer consisting of 0.04 mol/L citrate-phosphate buffer, pH 6.7, and 1% SDS, was prepared as follows: 1) Dissolve 2.1 g citric acid in 100 mL DIUF water to make 0.1 mol/L citrate buffer; 2) Dissolve 3.56 g sodium phosphate dibasic dihydrate in 100 mL DIUF water to make 0.2 mol/L phosphate buffer; 3) combine 36.4 mL 0.2 mol/L phosphate buffer and 13.6 mL 0.1 mol/L citrate buffer in a 100 mL volumetric flask and dilute to 100.0 mL to make 2x CPB; 4) dilute 50 mL 2x CPB and 5 mL 20% (w/v) SDS with 45 mL DIUF water; 5) the pH should be 6.7 ± 0.1 (if not, remake); 6) store at room temperature up to 6 mo.

Sample Preparation. PS 8670 (10 mg/mL in formulation buffer) was thawed from –80 °C to room temperature, inverted 3 to 5 times to homogenize vial contents, then maintained at 2 to 8 °C thereafter. For CE-SDS analysis under non-reducing conditions, 100 µg PS 8670 in 10 µL (or as indicated) was diluted with 85 µL of the indicated SDS sample buffer in Protein LoBind tubes

(Eppendorf, PN 89166-278 for 0.5 mL). To this was added 1 µL of 20% (w/v) SDS stock, 2 µL of 10 kDa internal standard, and 10 µL (or as indicated) of 0.5 mol/L iodoacetamide in water, prepared fresh. The sample was mixed by vortexing, then incubated for 5 min at 70 °C in a water bath (or as otherwise indicated). The sample was cooled to room temperature, vortexed, centrifuged briefly, and then transferred to sample vials for CE-SDS analysis.

For analysis under reducing conditions, 100 µg PS 8670 (in 10 µL) was diluted with 85 µL SDS sample buffer, 1 µL 20% (w/v) SDS, 2 µL 10 kDa internal standard protein, and 5 µL 2-mercaptoethanol. The sample was vortexed, then incubated for 10 min at 70 °C in a water bath, or as otherwise indicated. The sample was cooled to room temperature, and then vortexed, centrifuged, and transferred to an appropriate sample vial. The instrument qualification standard (IQ) was prepared by diluting 10 µL of MW Marker protein mix with 85 µL SDS sample buffer spiked with 2 µL 10 kDa internal standard. The sample was vortexed, and then incubated for 10 min at 70 °C. The sample was cooled to room temperature, vortexed, centrifuged, and transferred to a sample vial. Blanks were prepared by substituting formulation buffer in the place of PS 8670 in the appropriate volume.

Method Linearity/LOD/LOQ Sample Preparation. A dilution series of RM 8670 under reducing conditions was prepared in triplicate as follows. A stock solution (2.0 mg/mL) of reduced PS 8670 was prepared by diluting 70 µL 10 mg/mL RM 8670 with 250 µL citrate-phosphate/SDS sample buffer, 6 µL 10 kDa internal standard, and 16 µL 2-mercaptoethanol. A stock blank solution was prepared by mixing 140 µL L-His buffer, 12 µL 10 kDa internal standard, 32 µL 2-mercaptoethanol, and 500 µL citrate-phosphate/SDS sample buffer. The 2.0 mg/mL stock and the blank were vortexed and then incubated 10 min at 70 °C. After the samples cooled to room temperature, the PS 8670 sample was serially diluted using the blank sample to

yield the following dilution series: 1.5, 1.0, 0.5, 0.25, and 0.025 mg/mL. Samples (100 µL each) were analyzed by CE-SDS as described below in "Instrumental Method".

Preparation of Stressed Samples. PS 8670 (1 mg in 100 µL formulation buffer) was dispensed into a thin-walled polypropylene 0.2 mL PCR tube with cap (Fisher Scientific PN 14230205) and placed on a sheet of aluminum foil, reflective side up, in the chamber of a Stratalinker 2400 UV source equipped with 5 UVC bulbs (Eiko, F15T8/BL, 365 nm, 15 W). An identical sample was prepared, wrapped in aluminum foil to exclude light, and placed in the chamber as a thermal degradation control. The samples were irradiated for 21 h.

Data Analysis. Electropherograms were analyzed using the 32Karat software package (Sciex Separations) using optimized integration parameters (Tables S9, S10, and S11). Retention times and corrected peak areas were recorded for the non-reduced and reduced sample analysis as listed in Table S1.

Table S1 Measured and calculated assay results for CE-SDS analyses

Sample	Measurand Recorded	Calculated Quality Parameters
Blank	Presence of new peaks (Y/N)<br>10 kDa internal standard migration time (min)	n/a
IQ	Migration time (min) of ladder protein peaks: 10 kDa, 20 kDa, 35 kDa, 50 kDa, 100 kDa, 150 kDa, 225 kDa	n/a
8670 (non-reduced)	10 kDa internal standard migration time (min)<br>Monomer migration time (min)<br>Corrected area of monomer<br>Corrected area of fragment peaks: L, H, H:L, H:H, H:H:L, clip species	Monomeric purity (%)
8670 (reduced)	10 kDa internal standard migration time (min)<br>Migration time (min) of L and H<br>Corrected area of L, NGH, H, and thioether	Heavy chain relative abundance (%)<br>Light chain relative abundance (%)<br>Glycan occupancy (%)<br>Thioether relative abundance (%)

L = light chain; H = heavy chain; H:L = heavy chain:light chain fragment; H:H = heavy chain:heavy chain fragment; H:H:L = heavy chain:heavy chain:light chain fragment; NGH = aglycosylated/non-glycosylated heavy chain

Quality parameters were then calculated from recorded measurands using the equations below. Monomeric purity of 8670 was calculated according to equation S1:

$$Monomeric\ Purity(\%) = \frac{CA_{monomer}}{CA_{monomer} + \sum CA_{fragments}} \times 100\% \quad \text{(S1)}$$

Glycan occupancy of the heavy chain was calculated using equation S2:

$$Glycan\ Occupancy(\%) = \frac{CA_H}{CA_H + CA_{NGH}} \times 100\% \quad \text{(S2)}$$

Where H = heavy chain and NGH = aglycosylated/non-glycosylated heavy chain. Thioether relative abundance (RA) was calculated using equation S3:

$$Thioether\ RA(\%) = \frac{CA_{thio}}{CA_L + CA_H + CA_{NGH} + CA_{thio}} \times 100\% \qquad \text{(S3)}$$

Where thio = thioether, non-reducible species; H = heavy chain, L = light chain; and NGH = aglycosylated heavy chain.

A plot of IQ marker protein migration time versus the base 10 logarithm of nominal marker molecular weight was sometimes plotted for characterization purposes (see Figure S1 for representative electropherogram). This plot is not included in the quality parameters but may be useful in assay troubleshooting. Historical experience has been of an approximately linear curve, a linear fit of which has yielded R-squared values greater than or equal to 0.98 and relative residual standard deviations less than 5%.

Calculation of statistics including intermediate precision was performed in Microsoft Excel using the Analyse-it® plug-in (Analyse-it Software, Ltd., Leeds, UK) as discussed in [1]. Briefly, the precision for a given quality parameter was calculated by performing an ANOVA to estimate the total variance of the dataset and to model the components of the variance due to within-day variability (repeatability) and between-day variability (encompassing multiple columns, instrument drift, etc). This analysis was accomplished using the Analyse-it® measurement system analysis (MSA) precision tool and setting the model to “Y with 1 random factor”, where the factor was the date of analysis. The estimator was set to be standard deviation with a two-sided 95% confidence interval. The method was chosen to be “Exact/MLS”, and the “ANOVA” option was checked.

Method Linearity Regressions. Linear regression (LINEST function in Excel) of corrected area versus loading concentration was performed using each individual data point (as opposed to

means shown in Figure S2) to allow a statistical fit evaluation. Residuals were calculated based on the linear fit and residual standard deviation (rSD) and relative residual standard deviation (rrSD) were calculated for each fit. The rSD was calculated using Equation S4.

$$rSD = \frac{\sum(Y_{calc} - Y_{meas})^2}{n-2} \quad \text{(S4)}$$

where Y_{calc} is the theoretical Y value calculated from the line of best fit, Y_{meas} is the measured Y value, and n is the number of data points in the curve. The rrSD was calculated using Equation S5.

$$rrSD = \frac{rSD}{\sum Y_{meas}/n} \times 100\% \quad \text{(S5)}$$

Table S2 gives the goodness-of-fit parameters for each linear regression.

Table S2 Features of linear regression analysis of CE-SDS corrected area vs. concentration curves

Plot	R-Squared	rSD[a]	rrSD[b]	F Statistic[c]
Light Chain	0.998	160.6	2.6 %	7309.052
Heavy Chain	0.999	294.9	2.3 %	9877.871
Aglycosylated Heavy Chain	0.989	6.4	7.0 %	1199.495
Thioether	0.939	11.0	17.6 %	200.119

Analysis performed in triplicate at each of five concentration levels. [a]*rSD = residual standard deviation;* [b]*rrSD = relative residual standard deviation;* [c]$F_{critical}$ *=4.667 corresponding to an* α *= 0.05.*

Limit of Detection and Limit of Quantification. Limit of Detection (LOD) and Limit of Quantitation (LOQ) are calculated as follows. The 6-sigma signal-to-noise ratio (SNR) and relative abundance (RA) of the aglycosylated heavy chain (NGH) peak were recorded at the target loading concentration to be 9.1 (1.1) and 0.51 (0.03) % (SD), respectively. The aglycosylated heavy chain peak was chosen for this because it is at low abundance with a 6-sigma SNR close to the LOQ limit at the target loading concentration. The LOD and LOQ were calculated using Equations S6 and S7.

$$LOD(mg) = \frac{3}{SNR_{NGH}} \times \frac{RA_{NGH}(\%)}{100} \times C_{inj} \times V_{inj} \tag{S6}$$

$$LOQ(mg) = \frac{10}{SNR_{NGH}} \times \frac{RA_{NGH}(\%)}{100} \times C_{inj} \times V_{inj} \tag{S7}$$

where C_{inj} is concentration of total protein loaded in the experiment (mg/mL) and V_{inj} is the injection volume (mL). An electrokinetic injection was utilized for the CE experiments; therefore, V_{inj} (mL) was calculated based on equation S8.

$$V_{inj} = \mu_{mv} \pi r_{cap}^2 E_{EKI} t_{inj} \times 10^{-8} \tag{S8}$$

where μ_{mv} is the mean apparent mobility of the minor variant peak in $\frac{cm^2}{V \cdot s}$; r_{cap} is the internal radius of the capillary in µm; E_{EKI} is the electrokinetic injection field in V/cm; and t_{inj} is the EKI time in seconds. The apparent mobility of the aglycosylated heavy chain (mean of 15 measurements) was used for these calculations (μ_{mv} = 3.6 x10^{-5} $cm^2V^{-1}s^{-1}$). The mean apparent mobility was calculated using the following equation S9:

$$\mu_{mv} = \frac{l_d l_t}{V_{app} t_{mv}} \tag{S9}$$

where l_d is the capillary length to detector in cm; l_t is the total capillary length in cm; V_{app} is the separation voltage in volts; and t_{mv} is the migration time of the minor variant in seconds. The mass-based LOD and LOQ can be converted to a percent relative abundance corresponding to the experiment run at the target concentration using equations S10 and S11.

$$LOD(\%) = \frac{LOD}{C_{target} \times V_{inj}} \times 100 \tag{S10}$$

$$LOQ(\%) = \frac{LOQ}{C_{target} \times V_{inj}} \times 100 \tag{S11}$$

In the case of the CE assays discussed herein, C_{inj} = C_{target} because a minor variant (e.g. NGH) was present at appropriate SNR for this type of determination. This may not be true for all analytes and all assay types (as will be seen for SEC below). The two step calculation method described allows for mass-based LOD/LOQ to be calculated at a C_{inj} smaller than the C_{target} (but still within the linear range) and later converted to a percent-based LOD/LOQ at the target loading concentration (C_{target}) of the optimized assay.

Specificity. Method specificity and carryover was evaluated as described in the main text.

Intermediate Precision. For determination of intermediate precision, samples were prepared according to the optimized methods. Each day, a fresh vial of PS 8670 was thawed from ─80 °C to room temperature, inverted 5 times to mix, centrifuged briefly, and subjected to the appropriate sample preparation (reduced or non-reduced). One non-reduced sample preparation was performed per day for four days; this design was repeated for the reduced sample. Blank samples and instrument qualification (IQ) samples were prepared also as described. Samples were analyzed by CE-SDS as in

Table S3. The sequences were of the format Blank─IQ─(PS 8670 × 3)─IQ─Blank. Quality parameters were calculated and subjected to statistical analysis as described above.

Table S3 CE-SDS qualification injections

Capillary	Day	Number of PS 8670 Injections
Capillary # 1	1	3
	2	3
	3	3
Capillary # 2	4	3

Summary of Qualified CE-SDS Method

Optimized Sample Preparation. The optimized sample preparation method is summarized below. For analysis under non-reducing conditions and quantitation of monomeric purity:

1. Dilute 10 µL of 10 mg/mL mAb with 85 µL 0.04 mol/L citrate-phosphate/1% SDS sample buffer (pH 6.7) in a Protein LoBind tube.
2. Add 1 µL of 20 % (w/v) SDS and 2 µL of 10 kDa internal standard protein.

3. Prepare fresh 0.5 mol/L iodoacetamide by dispensing 600 µL DIUF water into the pre-weighed vial of iodoacetamide containing ≈56 mg of solid. Cap the vial and shake to dissolve.
4. Add 10 µL of 0.5 mol/L iodoacetamide to the vial of mAb. Vortex briefly to mix.
5. Incubate the mAb solution for 5 min in a 70 °C water bath.
6. Remove the vial from the water bath and allow to reach room temperature.
7. Briefly centrifuge the vial to collect any condensate.
8. Transfer the vial contents to a 0.2 mL sample vial and analyze by CE-SDS.

For analysis under reducing conditions and quantitation of glycan occupancy and thioether relative abundance:

1. Dilute 10 µL of 10 mg/mL mAb with 85 µL 0.04 mol/L citrate-phosphate/1% SDS sample buffer (pH 6.7) in a Protein LoBind tube.
2. Add 1 µL of 20 % (w/v) SDS and 2 µL of 10 kDa internal standard protein.
3. In the chemical safety cabinet, add 5 µL 2-mercaptoethanol to the tube. Cap tightly. Vortex briefly to mix.
4. Incubate the mAb solution for 10 min in a 70 °C water bath.
5. Remove the vial from the water bath and allow to reach room temperature.
6. Briefly centrifuge the vial to collect any condensate.
7. Transfer the vial contents to a 0.2 mL sample vial and analyze by CE-SDS.

Performance Criteria. The performance criteria for the method under non-reducing and reducing conditions were set for each parameter based on the measured intermediate precision. These criteria are useful for ensuring that the analytical method is in control, thus establishing confidence in the data acquired using the method. The criteria for the IQ are as follows:

- Visually conforms to expectation (expected peak shape and pattern)
- 10 kDa internal standard migration time falls within ±3u_c of the mean: (12.09 min to 12.81 min).
- 100 kDa marker peak migration time falls within ±3u_c of the mean: (21.34 min to 22.60 min).

The criteria for injections of PS 8670 under non-reducing conditions are as follows:

- Visually conforms to expectation (expected peak shape, no new peaks above LOD).
- 10 kDa internal standard migration time falls within ±3u_c of the mean: (12.09 min to 12.81 min).
- Monomer migration time falls within ±3u_c of the mean: (27.34 min to 29.04 min).
- Monomeric purity falls within ±3u_c of the mean: (97.65 % to 99.93 %).

The criteria for injections of PS 8670 under reducing conditions are as follows:

- Visually conforms to expectation (expected peak shape, no new peaks above LOD).
- 10 kDa internal standard migration time falls within ±3u_c of the mean: (12.09 min to 12.81 min).
- Light chain migration time falls within ±3u_c of the mean: (14.74 min to 15.84 min).
- Heavy chain migration time falls within ±3u_c of the mean: (18.58 min to 19.96 min).
- Light chain relative abundance falls within ±3u_c of the mean: (31.42 % to 32.62 %).
- Heavy chain relative abundance falls within ±3u_c of the mean: (66.66 % to 67.86 %).

Blank injections should contain only the 10 kDa internal standard peaks (main peak + known impurities) and no new peaks above the LOD.

CE-SDS Method Parameters

The detailed time programs for the CE-SDS instrument methods in 32Karat are given below in Tables S4-S6.

Table S4 CE-SDS capillary conditioning time program

	Time (min)	Event	Value	Duration	Inlet Vial	Outlet Vial	Summary	Comments
1		Rinse-Pressure	20.0 psi	10.00 min	BI:D1	BO:D1	forward	0.1 mol/L NaOH rinse to clean capillary surface
2		Rinse-Pressure	20.0 psi	5.00 min	BI:E1	BO:E1	forward	0.1 mol/L HCl rinse to neutralize capillary surface silanol group
3		Rinse-Pressure	20.0 psi	2.00 min	BI:F1	BO:F1	forward	Water rinse to remove acid residue
4		Rinse-Pressure	70.0 psi	10.00 min	BI:B1	BO:B1	forward	SDS-MW Gel Buffer rinse to fill the capillary
5	0.00	Separate-Voltage	15.0 kV	10.00 min	BI:C1	BO:C1	5.00 Min ramp, reverse polarity, both	SDS-MW Gel Buffer voltage equilibration

Table S5 CE-SDS high resolution separation time program

	Time (min)	Event	Value	Duration	Inlet Vial	Outlet Vial	Summary	Comments
1		Rinse-Pressure	70.0 psi	3.00 min	BI:D1	BO:D1	forward, In / Out vial inc 8	0.1 mol/L NaOH rinse to clean capillary surface-Automatic increment every 8 runs
2		Rinse-Pressure	70.0 psi	1.00 min	BI:E1	BO:E1	forward, In / Out vial inc 8	0.1 mol/L HCl rinse to neutralize capillary surface silanol group-Automatic increment every 8 runs
3		Rinse-Pressure	70.0 psi	1.00 min	BI:F1	BO:F1	forward, In / Out vial inc 8	Water rinse to remove acid residue-Automatic increment every 8 runs
4		Rinse-Pressure	70.0 psi	10.00 min	BI:B1	BO:B1	forward, In / Out vial inc 8	SDS Gel rinse to fill the capillary with SDS gel-Automatic increment every 8 runs
5		Wait		0.00 min	BI:A1	BO:A1	In / Out vial inc 8	DIUF, use for dipping to clean capillary tip-Automatic increment every 8 runs
6		Wait		0.00 min	BI:A4	BO:A4	In / Out vial inc 8	DIUF, use for dipping to clean capillary tip-Automatic increment every 8 runs
7		Inject – Voltage	5.0 kV	20.0 sec	SI:A1	BO:C1	Override, reverse polarity	Sample injection
8		Wait		0.00 min	BI:B4	BO:B4	In / Out vial inc 8	DIUF, use for dipping to avoid sample carry over - Automatic increment every 8 runs
9	0.00	Separate-Voltage	15.0 kV	35.00 min	BI:C1	BO:C1	1.00 Min ramp, reverse polarity, both, In / Out vial inc 8	SDS Gel for separation-Automatic increment every 8 runs
10	5.00	Autozero						

Table S6 CE-SDS capillary shutdown and storage time program

	Time (min)	Event	Value	Duration	Inlet Vial	Outlet Vial	Summary	Comments
1		Rinse-Pressure	70.0 psi	10.00 min	BI:D1	BO:D1	forward	0.1 mol/L NaOH rinse to clean capillary surface
2		Rinse-Pressure	50.0 psi	5.00 min	BI:E1	BO:E1	forward	0.1 mol/L HCl rinse to neutralize capillary surface silanol group
3		Rinse-Pressure	50.0 psi	2.00 min	BI:F1	BO:F1	forward	Water rinse to remove acid residue
4		Rinse-Pressure	70.0 psi	10.00 min	BI:B1	BO:B1	forward	SDS Gel rinse to fill the capillary
5	0.00	Separate-Voltage	15.0 kV	10.00 min	BI:C1	BO:C1	5.00 Min ramp, reverse polarity, both	SDS Gel for separation
6	10.00	Wait		0.00 min	BI:A1	BO:A1		Water used for capillary dip to prevent capillary from drying
7	10.00	Lamp – Off						

The initial conditions for the PA800 plus instrument used for all CE-SDS methods are given below in Tables S7 and S8.

Table S7 Initial conditions for CE-SDS methods

Parameter	Setting
Auxillary data channels	Current; max = 300.0 µA
Cartridge Temperature	25.0 °C
Sample Storage	25.0 °C
Peak detect threshold	2
Peak detect peak width	9
Trigger settings	Wait until cartridge coolant temperature is reached<br>Wait until sample storage temperature is reached
Analog output scaling factor	1
Inlet trays	Buffer: 36 vials<br>Sample: 48 vials
Outlet trays	Buffer: 36 vials<br>Sample: No tray

Table S8 PDA detector initial conditions for CE-SDS methods

Parameter	Setting
Electropherogram scan data	Acquisition enabled
Electropherogram scan data rate	2 Hz
Scan range	190 to 400 nm
Filter	Normal; Peak width: 16 to 25 points
Electropherogram Channel Data	Channel 1 enabled
Electropherogram channel data rate	2 Hz
Electropherogram channel	Wavelength = 220 nm; Bandwidth = 10 nm
Absorbance signal	Direct

The integration parameters employed for analysis of CE-SDS data in this work are given below in tables S9-S11.

Table S9 Integration parameters used for Linearity/LOD/LOQ data analysis

#	Event	Start Time	Stop Time	Value
1	Integration Off	0.000	11.000	0
2	Width	0.000	0.000	0.2
3	Threshold	13.000	60.000	50
4	Minimum Area	0.000	0.000	500

Table S10 Integration parameters used for analysis of non-reduced samples during qualification of CE-SDS

#	Event	Start Time	Stop Time	Value
1	Integration Off	0.000	11.000	0
2	Integration Off	31.000	35.000	0
3	Width	0.000	0.000	0.2
4	Threshold	0.000	14.000	2000
5	Threshold	14.000	22.500	50
6	Threshold	22.500	24.000	100
7	Threshold	24.000	35.000	50
8	Minimum Area	0.000	0.000	700
9	Shoulder Sensitivity	0.000	27.000	1e+006
10	Shoulder Sensitivity	27.000	28.500	1
11	Shoulder Sensitivity	28.000	35.000	1e+006

Table S4 Integration parameters used for analysis of reduced samples during CE-SDS qualification

#	Event	Start Time	Stop Time	Value
1	Integration Off	0.000	11.000	0
2	Width	0.000	0.000	0.2
3	Threshold	0.000	13.000	5000
4	Threshold	13.000	60.000	150
5	Minimum Area	0.000	0.000	1500
6	Shoulder Sensitivity	0.000	0.000	9999

SEC Materials and Methods

Buffer Preparation. Mass measurements for solution preparation were performed on a Metler Toledo AL54 balance. Formulation buffer (12.5 mmol/L L-histidine/12.5 mmol/L L-histidine HCl, pH 6.0) was prepared as described above for CE-SDS. Mobile phase buffer of the optimized assay consisted of 100 mmol/L sodium phosphate supplemented with 250 mmol/L sodium chloride (pH 6.8) and was prepared as follows: 1) Weight out 10.4042 g of sodium phosphate dibasic dihydrate, 4.9847 g of sodium phosphate monobasic, and 14.6027 g of sodium chloride and dilute with ≈950 mL of LC/MS water; 2) while recording pH with a calibrated pH meter, adjust pH by drop-wise addition of 1 mol/L sodium hydroxide or 1 mol/L hydrochloric acid to pH 6.80 ± 0.02; 3) transfer to a 1000 mL volumetric flask, rinse beaker with LC/MS water and adjust flask volume to 1000 mL using rinse water; 4) sterile filter using a 0.22 µm cellulose acetate membrane into a glass HPLC bottle; 5) Degas for 20 minutes prior to use and store at 2°C to 8°C.

SEC Method Development. The control point utilized for the central composite design (CCD) was 100 mmol/L sodium phosphate and 250 mmol/L sodium chloride (pH 6.8). The minimum and maximum (axial points of the CCD) concentrations for each factor were chosen to be between 20 % to 25 % and 70 % to 75 % of the absolute range of 25 mmol/L to 200 mmol/L for sodium phosphate and 0 mmol/L to 500 mmol/L for sodium chloride concentration. Using these ranges, the axial points chosen were 50 mmol/L and 150 mmol/L for sodium phosphate and 150 mmol/L and 350 mmol/L for sodium chloride. Table S12 shows the remaining calculated buffer concentration values.

Table S12 Calculated coordinates and concentration for SEC design of experiments central composite design buffer study (all concentrations in units of mmol/L and pH 6.8)

**(*x*,*y*) Value**	**Calculated *X* value (Sodium Phosphate)**	**Calculated *Y* value (Sodium Chloride)**
(0, 0)	100	250
(1,1)	135	320
(1, -1)	135	180
(-1, -1)	65	180
(-1, 1)	65	320
(0, α)	100	350
(α, 0)	150	250
(0, -α)	100	150
(-α, 0)	50	250

CCD Figures were prepared in Dataplot and various method performance metrics (resolution dimer and monomer (R_s) number of theoretical plates for the monomer (N), and monomer peak asymmetry (A_s) were fit with a quadratic model [2, 3]. The number of theoretical plates (column efficiency) of the monomer peak was determined using the statistical moment-based peak integration in Chromeleon 7 software instead of the USP integration method in order to fully monitor the peak tailing in calculation of the second peak moment (peak variance). The USP method (which uses a 5% peak height to evaluate peak fronting and tailing) was used as the default method for all other integration including determination of R_s and A_s.

Sample Preparation. Samples were removed from the −80°C freezer and placed on the bench to thaw. Samples were thawed at least 30 min at room temperature, and inverted 5 times with brief centrifuging in between each inversion to ensure homogeneity of the sample. Samples were then transferred to sample vial inserts and then to vials prior to analysis. Each sample was injected neat, with no dilution or buffer exchange. The concentration of each sample was

approximately 10 µg/µL. The instrument qualification standard (IQ) was prepared by reconstituting the contents of the vial in 0.5 mL DIUF water and gently mixed. The vial was placed in ice for several minutes and mixed again. The IQ standard was aliquoted into a micro-centrifuge tube and briefly centrifuged to remove any fine particulates before injecting onto the column. The IQ standard was stored at 2 ^{o}C to 8 °C and used within two weeks of preparation.

Method Linearity/LOD/LOQ Sample Preparation. The target concentration for SEC analysis is the neat, undiluted PS 8670 sample with no sample preparation required. Linearity was therefore assessed by injecting PS 8670 at varying injection volumes as opposed to manual dilutions. Injection values ranged from 1.8 µL to 10.2 µL, corresponding to 30% to 170% of the target load (18 µg to 102 µg).

Preparation of Stressed Samples. UV-Stressed Primary Standard 8670 was prepared as described above for CE-SDS.

Data Analysis. Raw chromatograms were processed with Thermo Scientific Dionex Chromeleon 7 Chromatography Data System using optimized integration parameters (Table S17). Retention times and peak areas were recorded for SEC sample analysis as listed in Table S13.

Table S13 Measured and calculated assay results for reducing SEC analyses

Sample	Measurand Recorded	Calculated Quality Parameter
Blank	Presence of new peaks (Y/N)	N/A
IQ	Presence of 5 main peaks (Y/N)<br>γ-globulin migration time (min)<br>Ovalbumin migration time (min)	N/A
8670	Monomer retention time (min)<br>Peak area of monomer, HMW, and LMW<br>Resolution (dimer-monomer)	Monomer relative abundance (%)<br>High molecular weight (HMW) relative abundance (%)<br>Low molecular weight (LMW) relative abundance (%)

A plot of IQ marker protein retention time versus the base 10 logarithm of nominal marker molecular weight was sometimes plotted for characterization purposes (see Figure S3 for representative chromatogram). This plot is not included in the quality parameters but may be useful in assay troubleshooting. Historical experience has been of an approximately linear curve, a linear fit of which has yielded R-squared values greater than or equal to 0.99 and relative residual standard deviations less than 1%.

Calculation of statistics including intermediate precision was performed in Microsoft Excel using the Analyse-it® plug-in (Analyse-it Software, Ltd., Leeds, UK) as discussed above in the CE-SDS section.

Method Linearity Regressions. Linear regression (LINEST function in Excel) of corrected area versus loading concentration was performed using each individual data point (as opposed to means shown in Figure S4) to allow a statistical fit evaluation. Residuals were calculated as discussed in the CE-SDS section above and are shown in Table S14.

Table S14 Features of linear regression analysis of SEC area vs. concentration curves

Peak	R-Squared	rSD[a]	rrSD[b]	F Statistic[c]
Trimer	0.991	0.01	4.5 %	1842
Dimer	0.999	0.04	1.9 %	11483
Monomer	0.999	1.18	0.6 %	138298
Fragment	0.992	0.02	4.8 %	2222.7

Analysis performed in triplicate at each of six concentration levels.[a]rSD = residual standard deviation; [b] rrSD = relative residual standard deviation; [c]$F_{critical}$ =3.634 corresponding to an α = 0.05.

Limit of Detection and Limit of Quantification. Limit of Detection (LOD) and Limit of Quantitation (LOQ) were calculated as follows. An 18 µg sample (n=3) of primary standard 8670 in formulation buffer (1.8 µL of 10 µg/µL PS 8670 solution), which corresponds to 30% of the target sample load was assessed. The limits of detection and quantification of the method were estimated from the signal-to-noise ratio (SNR) value of the fragment (LMW) peak number 5. The SNR and percent relative abundance of the LMW peak at 30 % of the target loading concentration were recorded to be 19.73 (1.45) and 0.135 (0.001) % (SD), respectively. Chromeleon software-reported SNR values were utilized, which are calculated using an equation of peak height/noise (mAU). The LOD and LOQ were calculated using Equations S6 and S7 (with LMW used in place of NGH). The mass-based LOD and LOQ were then converted to a percent relative abundance corresponding to the experiment run at the target loading concentration using equations S10 and S11.

Specificity. The method specificity with respect to potential matrix interferents and carryover was assessed as described in the main text. Percent recovery was determined from linearity injection data described. The extinction coefficient and absorbance at 280 nm are used

to predict the total mass of protein eluting from the column according to equation S15; and equation S16 allows an estimation of the recovery based on the known content of protein injected.

$$\mu g\ recovered = \frac{Area \times F}{\varepsilon \times l} \qquad \text{(S15)}$$

$$\%\ recovery = 100 \times \frac{\mu g\ recovered}{\mu g\ injected} \qquad \text{(S16)}$$

Where *F* is the flow rate (0.300 mL/min), ε is the extinction coefficient (1.42 mg/mL × cm at 280 nm), and *l* is the path length (cm). The total amount of "µg injected" was based on measured PS 8670 concentration of 10.014 mg/mL as determined in [1].

Table S15 Recovery values of PS 8670 at varying concentrations

% of Target Load	Total Protein (μg)	μg Recovered*	% Recovery[a] (Beers Law)
30	18	18.62 (0.56)	103.32 (0.95)
60	36	37.19 (0.10)	103.17 (0.28)
90	54	56.10 (0.33)	103.75 (0.61)
Target Concentration	60	62.19 (0.09)	103.51 (0.15)
120	72	74.61 (0.17)	103.48 (0.23)
150	90	93.94 (0.25)	104.23 (0.28)
170	102	106.86 (0.46)	104.62 (0.45)
		Mean	103.73
		Standard Deviation	0.52

[a]*Stated uncertainty represents one standard deviation (n=3).*

Intermediate Precision. The intermediate precision of the optimized method was estimated from 24 injections of PS 8670 over four columns and 8 days as described in Table S16. New buffer (100 mol/L sodium phosphate and 250 mmol/L sodium chloride, pH 6.8) was prepared for each day of analysis and a new vial of PS 8670 and instrument qualification (IQ) standard was also thawed each day; with the exception of days 1 and 2 where the same buffer and vial of material was used. These qualification days were originally part of the CCD method optimization experiments where preparation of new buffer and thawing of a new PS 8670 vial was not necessary. The injection sequence for days 3 to 8 followed the form: blank – IQ ×3 – blank – 8670 ×3 – blank – IQ – blank. The instrument qualification standard (IQ) was qualified simultaneously using day 3 to 8 data. Quality parameters were calculated and subjected to statistical analysis as described above.

Table S16 SEC qualification injections

Column	Day	Number of PS 8670 Injections
Column # 1	1	3
	2	3
Column # 2	3	3
	4	3
Column # 3	5	3
	6	3
Column # 4	7	3
	8	3

Summary of Qualified SEC Method

Performance Criteria. The performance criteria for the method were set for each parameter based on the measured intermediate precision. These criteria are useful for ensuring that the analytical method is in control, thus establishing confidence in the data acquired using the method. The criteria for IQ are as follows:

- Visually conforms to expectation (expected peak shape and pattern).
- The migration time of γ-globulin falls within the $\pm 3u_c$ of the mean: (3.665 min to 4.134 min).
- The migration time of ovalbumin falls within $\pm 3u_c$ of the mean: (4.398 min to 4.974 min).

The criteria for injections of PS 8670 are as follows:

- Visually conforms to expectation (expected peak shape and presence of 4 distinct peaks).
- Monomer relative abundance falls within $\pm 3u_c$ of the mean: (98.412 % to 99.154 %).

- High molecular weight and low molecular weight relative area fall within ±3u_c of the mean: (0.664 % to 1.376 %) and (0.172 % to 0.221 %) respectively.
- The migration time of the monomer falls within ±3u_c of the mean: (3.696 min to 4.196 min).
- The resolution between dimer and monomer falls within ±3u_c of the mean: (1.796 to 2.108).

Blank injections should contain no new peaks above the LOD.

The integration parameters employed for analysis of SEC data in this work are given below in Table S17.

Table S17 Integration parameters used for sample analysis during SEC qualification

Retention Time	Parameter Name	Parameter Value	Injection Type	Channel
Initial	Consider Void Peak	Off	Any	All Channels
Initial	Smoothing Width	Auto	Any	All Channels
0.000 [min]	Inhibit Integration	On	Any	All Channels
0.000 [min]	Minimum Area	0.000 [Signal*min]	Any	All Channels
1.636 [min]	Inhibit Integration	Off	Any	All Channels
5.468 [min]	Inhibit Integration	On	Any	All Channels

DLS Materials and Method

See main text for details.

Flow Imaging Materials and Methods

Buffer Preparation. To prepare the working buffer solution for the lyophilized polyclonal IgG, 1 tablet of phosphate buffered saline (PBS) (Sigma, St. Louis, MO) was placed into a volumetric flask containing 200 mL of deionized ultrafiltered, and sonicated for 10 minutes to mix the components. This buffer (composed of 0.01 mol/L phosphate buffer, 0.027 mol/L potassium chloride, 0.137 mol/L NaCl, pH 7.4) was filtered through a 0.22 µm PVDF filter. Ten milliliters of the filtered PBS buffer was added to the lyophilized polyclonal IgG to make a protein solution that was approximately 0.5 mg/mL. A micro-flea Teflon stir bar was added to the protein solution and stirred for 4 hours using a Model PC-161 laboratory stirrer (Corning, Tewksbury, MA) at 400 RPM to generate proteinaceous particles. After four hours of stirring, the aggregated solution was placed at 4 °C overnight.

Data analysis. Python 2.7 (Python Software Foundation, https://www.python.org/) was used to write a script that extracts raw data obtained from exported FI files and applies filters to remove edge particles, air bubbles, stuck particles, and particles smaller than 2 µm. Edge particles, specified by FI, were not considered in the analysis because they encounter the border of the image frame precluding accurate sizing of the particles. Air bubbles were defined as all particles with an equivalent circular diameter (ECD) greater than or equal to 5 µm and an aspect ratio greater than 0.9. Particles that possess both characteristics were removed by the script.

To remove stuck particles from the raw data, the field of view (1280 pixels by 1024 pixels) was divided into 10 pixels by 10 pixel bins to create a 2D histogram. Each bin value

represents the number of particles found in a given spatial location over the entire run. For a normal run, bin values vary from 0 to 5 particles per bin; bins with a stuck particle displayed a much higher particle number. If a bin contained a number of particles six standard deviations or more greater than the median of unique values in the histogram, all particles in that bin were rejected and were not included in the mass calculation. Once these filters were applied, the mass of the protein within the particles was calculated based on Equation S17 and S18 as described by Kalonia et al. [4]. The mass was calculated for each particle using the assumption that the density of dry protein was approximately 1.41 g/mL [5] and that 20% of protein particles were made up of protein (the remaining 80% was composed of water) [4]. The total mass of protein within all of the particles in a sample was obtained by summing the masses for each particle. The concentration of protein in the particles (Equation S18) was calculated by dividing the mass obtained from Equation S17 by the volume analyzed by the FI. Based on these two equations, it is possible to approximately describe how much protein was in the particles relative to the protein in solution. This was obtained by taking a ratio of the concentration of protein in the particles (Equation S18) to the total protein concentration in solution of the unstressed sample.

$$\text{mass(ng)} = \sum_{i=1}^{N}\left(\sqrt{1.70763-1.70864(1-C)^{0.45}-0.070638C}\right)\left(\frac{\pi}{30}\right)\left(\frac{1.41\ g/mL}{1000}\right)(ECD)^3 \quad \text{(S17)}$$

$$\text{Concentration of protein in particles (ng/mL)} = \frac{\text{mass(ng)}}{\text{volume analyzed(mL)}} \quad \text{(S18)}$$

C = circularity; represents the squate of the ratio of the circumference of an equivalent area circle over the measured perimeter of the particle (0 = most fibrous to 1 = spherical). *ECD* = equivalent

circular diameter (µm); defined as the diameter of a polystyrene microsphere with the same image area as the observed particle

Sample preparation for PS 8670. Four vials of the PS 8670 material were obtained for assessing precision of the flow imaging method. The PS 8670 samples were removed from the -80 °C freezer and placed at room temperature on the bench to thaw for thirty minutes. Prior to analysis, the vials were inverted gently 5 times.

Method Development and Optimization. The effect of degassing, effect of purge and prime volumes, effect of different solutions on the optimization step, and effect of sample handling on observed particle concentrations were assessed. For these studies, that required large volumes of samples, a commercially available polyclonal IgG solution, which was intentionally aggregated as described above, was used.

To study the effect of degassing, the polyclonal IgG sample was either analyzed on the instrument immediately or degassed for 10 minutes under vacuum prior to analysis. To study the effect of prime volume (volume required to clean the flow cell and tubing of the previous sample) on particle concentrations, the protein sample was analyzed as above (with no degassing), but either 2 mL or 0 mL of water was primed between each run. To study the effect of purge volume on particle concentration, the polyclonal IgG sample was analyzed with purge volumes of 0 µL, 30 µL, or 200 µL of water. Purge volume was composed of the sample and the flushing fluid remaining in the fluid path. This sample/fluid mix must be purged before sample analysis can begin. To study the effect on particle concentration of the fluid choice for instrument optimization (when the instrument determines the threshold pixel intensity to be used for particle isolation during analysis), each polyclonal IgG sample, prior to analysis, was optimized with either PBS buffer, or with the actual polyclonal IgG solution. To study the effect

of sample handling, two analysts measured the particle concentration in the aggregated polyclonal IgG samples. Prior to dispensing the sample for analysis, they either tilted the protein solution 5 times or swirled it for 10 s. All control experiments were performed in at least triplicates, unless otherwise noted.

The results from the method variation experiments are shown in Table S18. Particle concentration represents the cumulative particle concentration equal to or above a given equivalent circular diameter (*ECD*). The particle distribution is separated into the five size bins: ECD ≥ 2 µm, ≥ 4 µm, ≥ 8 µm, ≥ 12 µm, and ≥20 µm. The effect of degassing on subvisible particle concentration measurement is minimal (as indicated by little differences in the coefficient of variation (CV) values across the five bins, regardless of degassing). For the smaller particles, a 2 mL prime volume shows lower variability (lower CV) as compared to the polyclonal IgG samples that were run with no prime volume (prime volume = 0 mL). Using a purge volume of 30 µL prior to analyzing the polyclonal IgG solution shows the highest variability in concentrations of ECD ≥ 2 µm particles compared to the other two purge volumes tested. This volume was determined to be roughly equivalent to the volume of fluid that must pass from the top of the sample port to the point of analysis on the flow cell. When a purge volume of 200 µL or 0 µL was used, little differences in variability in concentration measurements were observed. Additionally, flow cell optimization with buffer or protein did not lead to significantly different results for most size bins. However, optimization using protein shows better reproducibility at counting larger particles and slightly worse repeatability at counting smaller particles. However, due to limited sample availability, it was determined that optimization with buffer or even water was the most convenient choice. From these studies, the

following variables were chosen to be used on the PS 8670 and RM 8671 analysis: no degas, prime volume = 2 mL, purge volume = 200 µL, optimization with water.

The results from the sample handling experiments are shown in Table S19. The bottom row (overall repeatability) represents the pooled data where the two analysts' results were combined to study the intermediate precision of concentrations in the different sized bins. The variability in concentration between the two analysts was not very different and can be seen by the relatively small CV values for the five size bins (ranging from 7% for the ≥ 2µm bin to 11% for the ≥ 20 µm bin). There were slightly higher variability within each analyst especially at higher size range but that could be because there were fewer larger particles present, leading to larger statistical variability.

Finally, to check if the PS 8670 material adsorbs onto the FI flow cell and tubing, a series of different solutions (buffers and protein) were run in a specific order to determine if adsorption was occurring, as shown in Table S20. Initially, the flow cell was purged with 10 mL of deionized ultrafiltered (DIUF) water to obtain a low particle count. After this, the histidine formulation buffer was run to obtain initial particle concentrations. Since detergents can readily remove adsorbed protein, 0.02% (w/v) of Tween 20 was added to the histidine buffer to aid in desorbing protein if adsorption was occurring. If the protein is not being adsorbed, particle concentrations in buffers will remain low, even after running the PS 8670 material. If protein adsorption is a problem, but the protein is readily washed off the surface of the flow cell or tubing with buffer, there will be an observable increase in particle concentration in the histidine buffer that is run after the PS 8670 material. If the protein is less likely to come off in buffer alone, a histidine buffer containing a small amount of detergent Tween (buffer + Tween) is run

after the PS 8670 sample. If the protein comes off in this solution, higher particle concentrations will be observed during the buffer + Tween run.

From Table S20, after run # 4 (PS 8670 run), the particle concentrations in all of the subsequent buffer runs are still low. There is a slight increase in concentration in run 6 (histidine + Tween) compared to the run # 3 (initial run of histidine + Tween). Similarly, after two more runs of PS 8670, the concentrations in the buffers (run # 8, 9, 11, and 12) are not significantly higher than their initial runs. There appears to be only a minor increase in subvisible particle concentrations in the buffers after the PS 8670 material has been run, indicating adsorption is not a problem.

Repeatability, Intermediate Precision, Count Accuracy, and Sizing Accuracy. The optimized method identified from the above section was used to assess the reproducibility, count accuracy, size accuracy, and precision that can be obtained with this technique. Count-Cal microspheres of nominally 5 µm diameter and 3000 mL^{-1} concentration were first vigorously shaken for 20 s and sonicated for 20 s. Then 0.7 mL was gently pipetted out and inserted into the sample port of the flow imaging system for analysis. Triplicates of these standards were analyzed each day over a period of 5 days. Initial Count-Cal concentration measurements were higher than manufacturer's specifications. A similar observation was made with multiple batches of the Count-Cal microspheres; this suggested that the FI flow cell was slightly larger than expected. To remedy this, a primary microsphere standard, composed of nominally 4 µm latex microspheres, was run multiple times on the instrument. These microspheres were tightly calibrated for concentration on a highly characterized light obscuration instrument. The experimental concentrations obtained on the DPA-4200 were compared to the precisely calibrated concentrations of the primary microsphere standard to obtain a concentration

correction factor. This correction factor was applied to all of the raw concentration data to adjust for the larger-than-expected flow-cell.

Suspensions of 2 µm and 10 µm polystyrene microspheres in DIUF water were also analyzed. ETFE particles, prepared as described above, were also analyzed in triplicates over a period of three days. For every sample analyzed in all of these studies (unless otherwise noted), the FI optimize illumination step was performed with water, and 0.7 mL of the sample was loaded into the FI [6]. Of this 0.7 mL, 0.2 mL was used for purging the instrument at a flow rate of 0.17 mL/min. Between each run, 2 mL of water was used for priming at a flow rate of 6 mL/min.

The results of these studies are shown in Table S21. Over the span of 5 days, the Count-Cal solution, corrected for FI cell thickness, showed little variability with a mean particle concentration of 3095 (87) mL^{-1} (SD) between 3 µm and 8 µm in size. The intra-day measurements ranged from 2977 mL^{-1} to 3128 mL^{-1} with the CV values fluctuating from 1% to 4%, which is within the acceptable range of the FI (concentration repeatability is ± 5%, according to the instrument manual). The concentration accuracy prescribed by the instrument manufacturer is ± 10% with the Count-Cal bottle label stating that the concentration should fall in the range 3000 mL^{-1} ± 300 mL^{-1}. The particle size distribution for each sample was analyzed to determine the mode value in size in the samples; all size measurements are in ECD. The sizing of the nominally 5 µm microspheres ranged from 4.89 µm to 4.96 µm, with the CVs ranging from almost 0% to 3%, which is within the acceptable range (sizing repeatability = ± 5%) as given by the instrument manual. The Count-Cal bottle label states that the microspheres have a mean diameter of 5.010 µm ± 0.035 µm in size. The observed size is within the manufacturer's specifications.

The 2 µm and 10 µm microspheres were also run to study the precision in the concentration and size measurements. The concentration measured for both microsphere sizes was reproducible over three runs, with CVs below or equal to 2%. The microspheres were sized accurately and precisely (1.88 µm and 9.64 µm) with low CV values (≤ 0.5%). According to the instrument manual, sizing accuracy for spherical polystyrene microspheres is ± 0.5 µm for particles < 5 µm, and ±5 % for particles ≥ 5 µm.

Similarly, even when irregular shaped particles, such as ETFE, were assessed for particle concentrations over three days in five distinct size bins (ECD ≥ 2 µm, ≥ 4 µm, ≥ 8 µm, ≥ 12 µm, and ≥ 20 µm) the results were reproducible. Variabilities (inter-day and intra-day) in concentrations were minimal and intra-day CVs were similar to the inter-day CVs. For the ≥ 2 µm bin, the CVs, for both inter-day and intra-day runs, were not greater than 4%. For ≥ 4 µm bin, the CVs were ≤ 4%; ≤ 6 % for the ≥ 8 µm bin; ≤ 7% for the ≥ 12 µm bin; and ≤ 8 % for the ≥ 20 µm sized particles.

Table S18 Effect of method variations on repeatability of sizing and counting of subvisible particles in an aggregated polyclonal IgG solution. The concentrations of the polyclonal IgG particles are separated into five size bins (ECD ≥ 2 μm, ≥ 4 μm, ≥ 8 μm, ≥ 12 μm, ≥ 20 μm). Each value is a mean of at least 3 separate runs (n = 3) with the uncertainty expressed as (SD).

Method Variations											
		ECD ≥ *2 μm*		ECD ≥ *4 μm*		ECD ≥ *8 μm*		ECD ≥ *12 μm*		ECD ≥ *20 μm*	
		Particle Concentration (mL^{-1})	CV (%)	Particle Concentration (mL^{-1})	CV (%)	Particle Concentration (mL^{-1})	CV (%)	Particle Concentration (mL^{-1})	CV (%)	Particle Concentration (mL^{-1})	CV (%)
**Degas**	No Degas	116518 (6441)	6	16684 (348)	2	4302 (118)	3	2071 (66)	3	747 (26)	4
	10 min Degas	125246 (10931)	9	17325 (933)	5	4140 (98)	2	1983 (77)	4	726 (18)	3
**Prime Volume**	2 mL	116517 (6441)	6	16684 (348)	2	4302 (118)	3	2071 (66)	3	747 (26)	4
	0 mL	98141 (12261)	12	16354 (478)	3	4316 (30)	1	2082 (4)	0	742 (49)	7
**Purge Volume**	200 μL	116518 (6441)	6	16684 (348)	2	4302 (118)	3	2071 (66)	3	747 (26)	4
	30 μL	105343 (21567)	20	15989 (1158)	7	4157 (42)	1	1996 (12)	1	735 (3)	0
	0 μL	107010 (5213)	5	15522 (417)	3	3945 (49)	1	1925 (40)	2	742 (33)	4
**Optimization**	Buffer	99763 (5332)	5	15993 (133)	1	4250 (155)	4	2058 (135)	7	750 (95)	13
	Protein	108241 (8410)	8	16843 (162)	1	4430 (118)	3	2184 (147)	7	808 (66)	8

Table S19 Effect of sample handling on the repeatability of sizing and counting of subvisible particles in an aggregated polyclonal IgG solution. The concentrations of the polyclonal IgG particles are separated into five size bins (ECD ≥ 2 μm, ≥ 4 μm, ≥ 8 μm, ≥ 12 μm, ≥ 20 μm). Each value is a mean of at least 3 separate runs (n = 3) with the uncertainty expressed as (*SD*). Intermediate precision is the pooled results from Analyst 1 and Analyst 2.

Sample Handling											
		ECD ≥ *2 μm*		ECD ≥ *4 μm*		ECD ≥ *8 μm*		ECD ≥ *12 μm*		ECD ≥ *20 μm*	
		Particle Concentration (mL^{-1})	CV (%)	Particle Concentration (mL^{-1})	CV (%)	Particle Concentration (mL^{-1})	CV (%)	Particle Concentration (mL^{-1})	CV (%)	Particle Concentration (mL^{-1})	CV (%)
**Analyst 1: IgG**	Tilt 5X	14267 (1133)	8	2588 (13)	0	797 (22)	3	374 (30)	8	146 (3)	2
	Swirl 10 s	15882 (699)	4	2493 (107)	4	741 (66)	9	334 (39)	12	133 (13)	10
**Analyst 2: IgG**											
	Tilt 5X	16067 (436)	3	2656 (71)	3	823 (24)	3	381 (5)	1	141 (25)	18
	Swirl 10 s	15875 (998)	6	2692 (68)	3	796 (32)	4	388 (13)	3	153 (15)	10
**Intermediate Precision**	Tilt & Swirl	15523 (1057)	7	2607 (101)	4	789 (46)	6	369 (31)	8	143 (16)	11

Table S20 Assessing the impact of protein adsorption of PS 8670 on the concentration of subvisible particles detected by the flow imaging method. Each sample (DIUF water; Buffer = histidine buffer; Buffer + Tween 20 = histidine buffer + 0.02% (w/v) Tween 20) was run in the order as shown below.

**Order of Runs**	**Samples**	***Particle Concentration*** **(ECD ≥ 2 µm) (mL^{-1})**
1	DIUF water	2
2	Buffer	4
3	Buffer + Tween 20	4
4	PS 8670 n1	2066
5	Buffer	4
6	Buffer + Tween 20	19
7	PS 8670 n2	1620
8	Buffer	29
9	Buffer + Tween 20	15
10	PS 8670 n3	4776
11	Buffer	25
12	Buffer + Tween 20	4

Table S21 Repeatability measurements of 5 µm Count-Cal, 2 µm polystyrene microspheres, 10 µm polystyrene microspheres, and ETFE particles. The concentrations of the ETFE particles are separated into five size bins (*ECD* ≥ 2 µm, ≥ 4 µm, ≥ 8 µm, ≥ 12 µm, ≥ 20 µm). Each value is a mean of at least 3 separate runs (*n* = 3) with the uncertainty expressed as (SD).

**Repeatability Measurements**										
**Count-Cal - 5 µm**	*Conc. Precision*		*Sizing Precision*							
	Particle Concentration (mL^{-1})	CV (%)	Mean size (µm)	CV (%)						
Day 1	2977 (35)	1	4.94 (0.13)	3						
Day 2	3181 (72)	2	4.96 (0.14)	3						
Day 3	3123 (97)	3	4.89 (0)	0						
Day 4	3065 (40)	1	4.89 (0)	0						
Day 5	3128 (55)	4	4.89 (0)	0						
Inter-day	3095 (87)	3	4.91 (0.09)	2						
**2 µm polystyrene microspheres**	11784 (134)	1	1.88 (0)	0						
**10 µm polystyrene microspheres**	19003 (356)	2	9.64 (0.05)	0.5						
**ETFE**	ECD ≥ 2 µm		ECD ≥ 4 µm		ECD ≥ 8 µm		ECD ≥ 12 µm		ECD ≥ 20 µm	
	Particle Concentration (mL^{-1})	CV (%)	Particle Concentration (mL^{-1})	CV (%)	Particle Concentration (mL^{-1})	CV (%)	Particle Concentration (mL^{-1})	CV (%)	Particle Concentration (mL^{-1})	CV (%)
Day 1	39407 (571)	1	25261 (171)	1	13353 (67)	0	7553 (180)	2	2893 (58)	2
Day 2	39659 (1100)	3	24805 (1026)	4	12929 (778)	6	7279 (483)	7	2887 (228)	8
Day 3	40193 (995)	3	24763 (924)	4	12771 (541)	4	7140 (200)	3	2854 (3)	0
Inter-day	40147 (1491)	4	25109 (867)	4	13003 (513)	4	7251 (387)	5	2838 (170)	6

Supplemental Figures

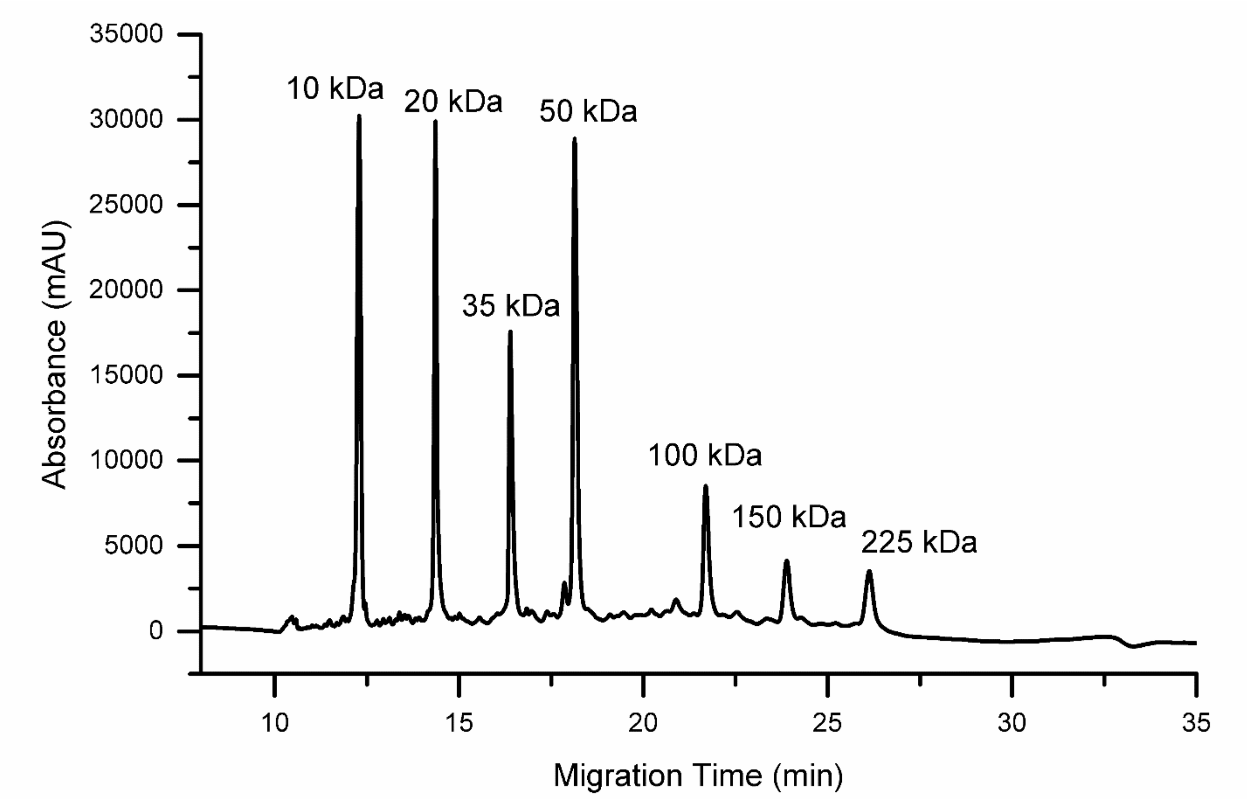

Fig. S1 Representative electropherogram of the CE-SDS instrument qualification (IQ) standard. Labels indicate the nominal molecular weight (MW) of each peak

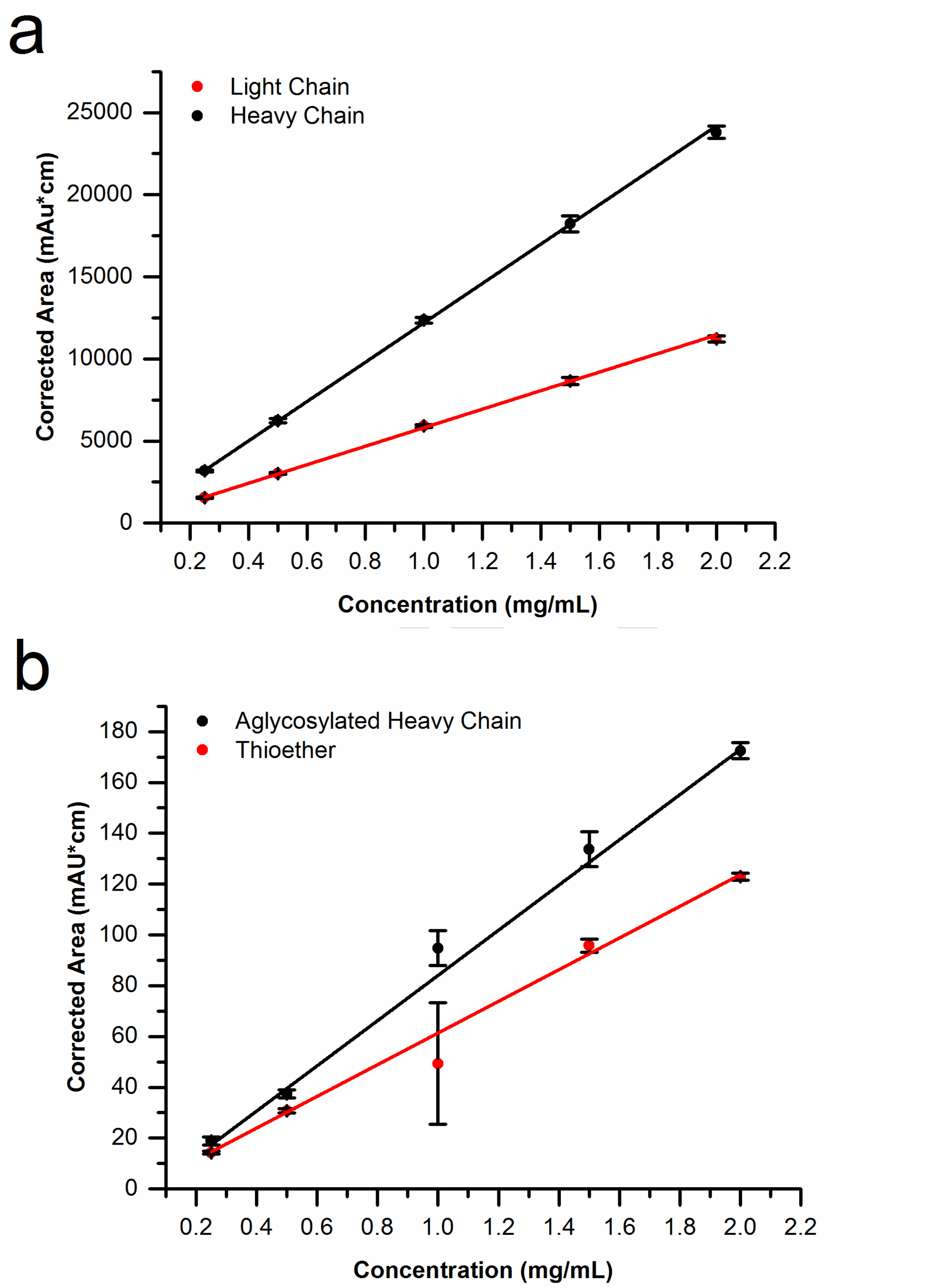

Fig. S2 Linearity of CE-SDS (reducing) assay response for (a) heavy chain and light chain (b) Aglycosylated heavy chain and thioether over a range of PS 8670 concentrations. Error bars represent SD (n = 3)

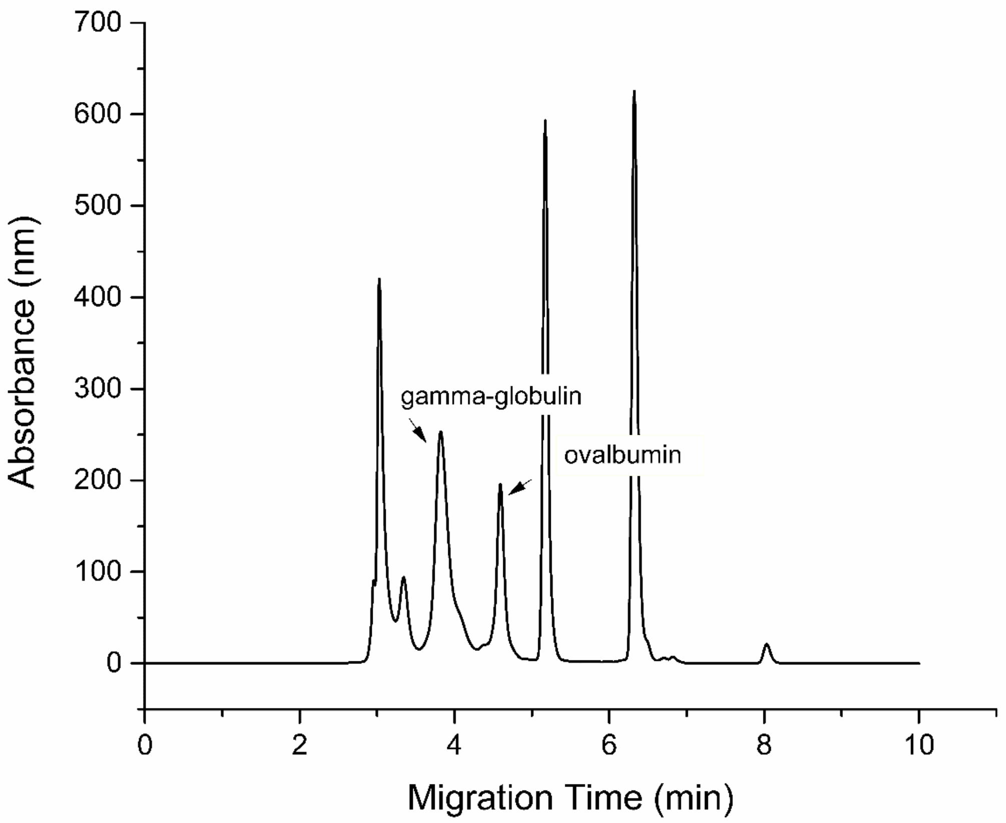

Fig. S3 Representative chromatogram of the SEC instrument qualification (IQ) standard. Labels indicate the component of the IQ standard used to set IQ performance criteria

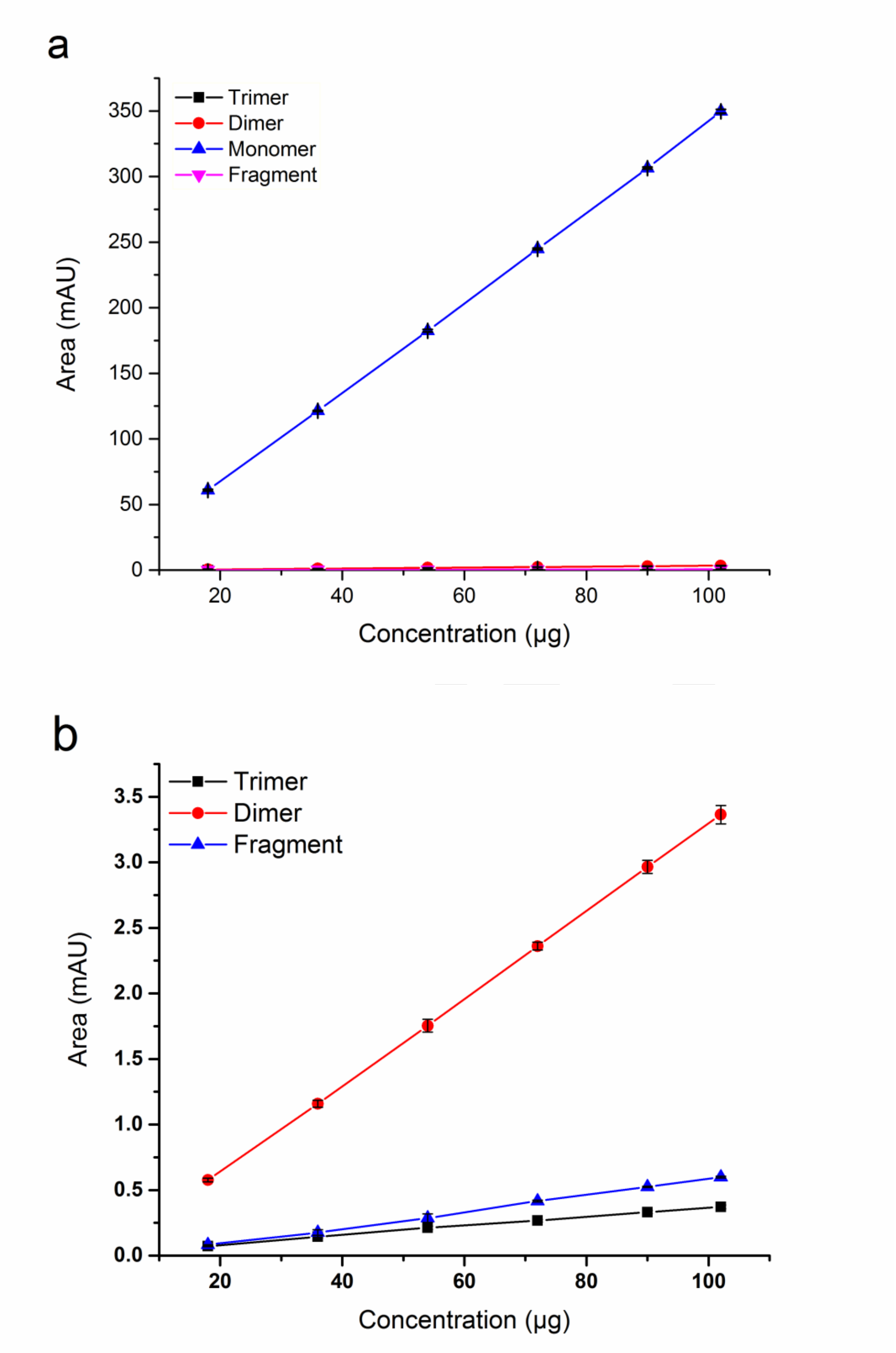

Fig. S4 (a) Linearity of Primary Standard 8670 for all peaks (trimer, dimer, monomer and fragment. (b) is zoomed in along the Y-axis to show scaling of trimer, dimer, and fragment area. Error bars represent SD (n=3)

1. Schiel JE, Turner A. The NISTmAb Reference Material 8671 Lifecycle Management and Quality Plan. Anal Bioanal Chem. 2018. doi:10.1007/s00216-017-0844-2.
2. Filliben JJ. DATAPLOT- an interactive high-level language for graphics, non-linear fitting, data analysis, and mathematics. SIGGRAPH Comput Graph. 1981;15(3):199-213. doi:10.1145/965161.806807.
3. Heckert NA. Dataplot website at the National Institute of Standards and Technology. 2001. http://www.itl.nist.gov/div898/software/dataplot.html/. 2016.
4. Kalonia C, Kumru OS, Prajapati I, Mathaes R, Engert J, Zhou S et al. Calculating the mass of subvisible protein particles with improved accuracy using microflow imaging data. J Pharm Sci. 2015;104(2):536-47. doi:10.1002/jps.24156.
5. Zolls S, Gregoritza M, Tantipolphan R, Wiggenhorn M, Winter G, Friess W et al. How subvisible particles become invisible-relevance of the refractive index for protein particle analysis. J Pharm Sci. 2013;102(5):1434-46. doi:10.1002/jps.23479.
6. Sharma DK, Oma P, Pollo MJ, Sukumar M. Quantification and characterization of subvisible proteinaceous particles in opalescent mAb formulations using micro-flow imaging. J Pharm Sci. 2010;99(6):2628-42. doi:10.1002/jps.22046.
